# Childhood temperament and adulthood personality differentially predict life outcomes

**DOI:** 10.1038/s41598-022-14666-0

**Published:** 2022-06-18

**Authors:** Amanda J. Wright, Joshua J. Jackson

**Affiliations:** Washington University in St. Louis, St. Louis, USA

**Keywords:** Psychology, Human behaviour

## Abstract

Debate has long surrounded whether temperament and personality are distinct sets of individual differences or are rather two sides of the same coin. To the extent that there are differences, it could indicate important developmental insights concerning the mechanisms responsible for linking traits with outcomes. One way to test this is to examine the joint and incremental predictive validity of temperament and personality in the same individuals across time. Using a longitudinal sample spanning 3 decades starting at infancy and followed up to 37 years old (*N* = 7081), we ran a series of Bayesian generalized linear models with measures of childhood temperament and adult-based personality to predict outcomes in several life domains. Results indicated that while each set of individual differences were often related to the same outcomes, there were instances in which temperament provided incremental validity above adult personality, ranging from 2 to 10% additional variance explained. Personality in childhood explained the most variance for outcomes such as cognitive ability and educational attainment whereas personality performed best for outcomes such as health status, substance use, and most internalizing outcomes. These findings indicate childhood and adulthood assessments of personality are not redundant and that a lifespan approach is needed to understand fully understand life outcomes.

Debate has historically surrounded whether childhood and adult personality are two sides of the same coin^1–3^, but despite the history of controversy, evidence indicates there is considerable overlap in childhood and adulthood personality traits^4,5^. If it were the case that adult personality is merely a later form of personality in childhood, one would reasonably expect an individual’s earlier temperament and later personality to predict similar life outcomes and/or show redundant predictive validity. However, if there are predictive differences, this would point to a number of developmental insights concerning the mechanisms responsible for linking individual differences with life outcomes.

While childhood temperament does predict later adult outcomes^6–8^, it is unclear whether child and adult assessments are redundant in the prediction of life outcomes as no study has directly examined this question. Using a large-scale representative sample over 30 years, we directly test the incremental predictive validity of childhood temperament above later adult-based personality to assess the uniqueness between the two types of age-graded individual differences.

## Does it matter when we assess personality?

Given that personality is moderately consistent across the lifespan^9,10^, it is important to identify *when* personality is most important. Child and adult personality prediction of life outcomes can yield a number of patterns, each suggesting different mechanisms linking personality with life outcomes.

First, the “it doesn’t matter when” pattern describes that if one wanted to predict outcomes with personality, any assessment across the lifespan would suffice. If childhood and adult personality traits equivalently predict future life outcomes, this would suggest the mechanisms linking traits measured at distinct points of individuals’ lives operate similarly and, ultimately, yield equal predictions of future outcomes. In support of this perspective, both childhood and adulthood personality assessments predict similar outcomes^5,6,8,11^.

A second possible pattern of associations is “all that matters is where you end up.” Whereas the previous pattern emphasizes the redundancy of assessments, this perspective suggests the strongest associations for assessments of personality are those closest in time to the outcomes they are trying to predict. As with any developmental processes, time introduces noise into the system. The result of this introduction of noise is that it continually builds and accumulates. This error generating process is (partly) the reason why decades-long longitudinal associations are weaker and harder to come by than associations closer in time^12^. This perspective puts emphasis on adulthood, and subsequently neglects childhood, as being relevant to understanding adult life outcomes such as health, wealth, and well-being.

The third pattern is the “it’s where you start *and* finish.” In contrast to the above pattern, this third perspective is that of a lifespan approach. It emphasizes that it is necessary to understand who an individual is throughout their entire life to best understand their current and future development. With this approach in mind, it becomes readily apparent that childhood personality is a rich source of individual differences that are inextricably related to an individual’s status in life at any point in time.

Importantly, past studies provide a reasonable basis for expecting child and adult personality to uniquely predict outcomes. This notion is supported in part by the fact that although there is nonzero stability from childhood to adult personality, these associations tend to be modest^10^, thus allowing for within- and between-person change. Hill et al.^13^ outlined three overlapping processes that serve as potential mechanisms by which childhood personality differentially predict future adult outcomes compared to adult-based personality measures.

First, the opportunities and snares hypothesis suggest that there are child-relevant events and situations directly associated with adult outcomes^13^. Personality at this time is important because one cannot make up for lost time if these opportunities are passed. Childhood personality plays an important role in developmental branching such that it predisposes them to take certain paths in life. Taking certain paths early in life restricts the ease of or ability to take other paths later in life, which emphasizes the widespread, downstream consequences of this early-life branching. For example, children who score higher in effortful control tend to do better and work harder in school^14,15^. These children are then more likely to obtain higher levels of education^16^, which itself predicts other future positive outcomes. In comparison, children who score lower on related traits are more likely to engage in substance use^17,18^, which itself predicts even more frequent substance use and other delinquent behaviors in adulthood^19^.

Second, the differential maturation hypothesis posits that the trajectories and rates of personality development and change experienced prior to adulthood can affect future outcomes^13^. At the core of this idea lies individual differences in rates of change during childhood. If people change at different rates, then having multiple assessments of a construct is important. Third, the differential pathways hypothesis describes those pathways that explain why personality affects future success may differ across the lifespan^13^. For example, it might be expected that the effects of personality on income are mostly driven by adult personality trait levels (e.g., working productively versus counterproductive work behaviors^20,21^). However, the paths linking personality and financial success may begin much earlier in life such as through greater educational attainment.

## Current study

In a longitudinal study of more than 7000 individuals assessed from birth to adulthood, we predicted outcomes in several life domains (e.g., health, relationships, career) using multi-method assessments of child and adult personality measured upwards of 30 years apart. We address two key questions, both from the lens of explaining variance in outcomes: (1) does temperament predict outcomes in adulthood and (2) does childhood temperament predict outcomes above and beyond adult-based personality? It should be noted that the terms “temperament” and “personality” are sometimes used interchangeably in the literature. For simplicity, we use the term “temperament” to refer to the assessment of individual differences in childhood. However, at its core, temperament reflects individual differences in children’s behavior and tendencies, which is consistent with the traditional definition of personality^5^. Thus, we ultimately consider this manuscript to be a test of the incremental predictive validity of childhood personality relative to adulthood personality.

## Methods

### Participants

Participants consisted of 7081 individuals from the National Longitudinal Survey of Youth 1979—Child and Young Adult (NLSY79-CYA) sample. The National Longitudinal Survey of Youth 1979 (NLSY79) is an ongoing longitudinal study conducted by the U.S. Bureau of Labor Statistics (BLS). The NLSY79 began in 1979 and consisted of a (then) nationally representative sample of 12,686 men and women who were all 14 to 21 years of age^22^. As of 2018, the women of the NLSY79 were between the ages of 53 to 62 and there were 11,545 children born to the NLSY79 mothers. The NLSY79-CYA sample consists of the offspring of the original mothers of the NLSY79 sample.

Across all waves, ages ranged from infancy (0 years old) to 37 years (*M* = 15.24, *SD* = 8.78). The average age in our sample at the final measurement occasion was 27.73 years old (*SD* = 4.87, *Min* = 15, *Max* = 37). Among participants, 39.4% of the sample identified as white (*N* = 2792), 36.2% as Black (*N* = 2564), 23.4% as Hispanic/Latinx (*N* = 1658), and 1.0% other (*N* = 67). There were 3594 males (50.8%) and 3487 females (49.2%). The last wave of data in our study was collected in 2016.

Participants were included in the present study if they had measures of childhood temperament *and* adult-based personality. Since this is a large panel study, participants who complete one measure are expected to have data for other measures at the same timepoint (i.e., if participants had personality data, they also had outcome data). Thus, attrition analyses were conducted that compared individuals who *only* had temperament data versus those who were included in the present study (i.e., had temperament and adult-based personality data). Compared to individuals included in our study (*N* = 7081), participants who only had temperament data (*n* = 2039) scored lower on fearfulness (*t*(1454) = 3.60, *p* < 0.001, *d* = − 0.12), higher on insecure attachment (*t*(2567.1) = − 3.88, *p* < 0.001, *d* = 0.11), and lower on sociability (*t*(2289.7) = 4.15, *p* < 0.001, *d* = − 0.12). Additionally, participants who only had temperament data, compared to those in our study, included a larger proportion of White participants (χ^2^(1) = 409.95, *p* < 0.001), a smaller proportion of Black participants (χ^2^(1) = 260.10, *p* < 0.001), a smaller proportion of Hispanic participants (χ^2^(1) = 26.88, *p* < 0.001), and had lower education levels (*t*(581.16) = 5.64, *p* < 0.001, *d* = − 0.97).

### Measures

#### Childhood temperament

Temperament was assessed in children ages 0 to 6 (*M* = 3.76, *SD* = 2.01) using scales adapted from the Infant Behavior Questionnaire^23^, compliance scale^24^, and additional items selected by one of the creators of the compliance scale (Joseph Campos). Participants in our study provided data for the temperament traits of activity, fearfulness, positive affect, and predictability from ages 0–11 months and for compliance and insecure attachment from ages 12–83 months^25^. All were maternal report. Then, sociability was assessed across the years with three items answered by the interviewer. Average Cronbach’s alpha values were 0.69 or greater. The number of waves of data for any temperament dimension ranged from 1 to 4; 1096 participants had 1 wave, 1207 had 2 waves, 2917 had 3 waves, and 1861 had 4 waves. The temperament qualities had an average prediction interval of nearly 25 years with a max of over 30 years.

#### Personality

Personality was assessed using the Ten Item Personality Inventory (TIPI^26^) in adolescents and adults (*M*_age_ = 23.04, *SD*_age_ = 4.93, *Min*_age_ = 15, *Max*_age_ = 35) up until 2014. This measure assesses the Big Five personality traits^26,27^. The number of waves for personality ranged from 1 to 4; 729 participants had 1 wave, 1941 had 2 waves, 3604 had 3 waves, and 807 had 4 waves.

#### Outcomes

##### Health

Included outcomes in the health domain include self-report health status and body mass index (BMI). Health status was assessed with a single-item measure asking, “How would you describe your present health?” and treated as an ordinal variable. Response options were on a Likert scale consisting of 1 (poor), 2 (fair), 3 (good), 4 (very good), and 5 (excellent). The last available wave of data for these variables were used as the outcome for each participant. BMI was calculated from the height and weight variables for each participant, standardized, and treated as continuous.

##### Internalizing

Included outcomes included diagnoses of anxiety and depression; record of ever seeing a counselor for emotional, behavioral, or mental problems; and record of ever attempting suicide. The variables were coded such that 1 indicated a response of “yes” during any available waves for a single participant and 0 indicated a response “no” at every wave (i.e., dummy-coded).

##### Externalizing

Included outcomes were a diagnosis of attention deficit hyperactivity disorder (ADHD ), reported number of substances used across all available waves for a participant, and ever going to jail. For substance use, items asking if the participant had ever used one of eight substances (alcohol, cigarettes, cocaine, hallucinogens, marijuana, downers, inhalants, stimulants) were used to create a variable for the number of substances the individual has done. The variables for an ADHD diagnosis and ever going to jail were dummy-coded.

##### Cognition

Variables assessing cognitive performance consisted of a total score of a forwards and backwards digit span count, word recall, Peabody Individual Achievement Test (PIAT) math assessment, PIAT reading comprehension assessment, and PIAT reading recognition assessment^28^. Raw summary scores for each cognitive assessment were obtained directly from the NLSY Investigator database. Final cognition variables were standardized and treated as continuous.

##### Relationships and family

Outcomes in the relationship domain included relationship satisfaction at the last available wave for a participant, record of ever being married, ever being divorced, number of marriages, and ever having children. The variables for ever being married, divorced, or having children were dummy-coded. There were three possible variables for relationship satisfaction, each asking about satisfaction with a different type of relationship (boyfriend/girlfriend, partner, spouse). Since participants did not have data for more than one variable at a given wave (as they could not have a girlfriend/boyfriend AND a spouse, for example), these three items were combined to form a single relationship satisfaction variable and was treated as ordinal.

##### Education, career, and financial

Included variables were highest degree obtained by the participant, being employed at the wave following their last personality assessment, median annual salary, and record of ever being the recipient of government financial assistance (i.e., welfare). Highest degree obtained was treated as an ordinal variable and its value was determined by the highest value across all available waves for a participant. Being employed and ever receiving welfare were dummy-coded. Median annual salary was calculated across all available waves for a participant, standardized, and treated as continuous.

##### Civic engagement

Included variables were being religious and volunteering. The variables were dummy-coded.

#### Control variables

Variables that have been previously used in past studies and that were of theoretical and practical relevance were included to account for potentially influential differences surrounding birth and early childhood of the participants. These variables were age at the last wave of the outcome variable, gender (male = 0, female = 1), race, mother’s age at birth, whether or not the child was breastfed, number of weeks the mom was pregnant with the participant, child’s height and weight at birth, whether the mother reported any drinking or smoking during pregnancy, and mother’s highest education level.

### Transparency and openness

Within this methods section, we report how we determined our final sample size through inclusion criteria, all measures used along with their psychometric properties, and we follow the APA Style Journal Article Reporting Standards (JARS^29^). Data are freely accessible at https://www.nlsinfo.org/investigator and code for all data cleaning and analyses is available at https://osf.io/kyrq7/. The Institutional Review Board (IRB) at Washington University in St. Louis deemed this project exempt from IRB approval because it involves accessing a publicly available dataset and thus does not meet federal definitions under the jurisdiction of an IRB (ID#: 202107190). The APA’s ethical standards for conducting research were followed throughout the duration of this study. Data were analyzed using R, version 4.0.3^30^ and the package brms^31^. This study’s design and its analyses were not pre-registered.

### Analysis plan

Bayesian generalized linear regressions were conducted for each outcome with (a) all temperament dimensions, (b) all personality traits, and (c) all temperament and personality entered simultaneously as predictors. All temperament and personality variables were standardized to aid in interpretation and model convergence. To calculate our primary parameter of interest—the incremental R^2^ values—all models were first fit without covariates. Then, only for the purpose of obtaining individual trait estimates that may be of interest (i.e., calculating the incremental R^2^ for the temperament models was no longer needed), models including covariates were fit. Priors were weakly regularizing and centered around 0. Binomial distributions were used for any dichotomous outcome variables; cumulative distributions were used for ordinal variables; Poisson distributions were used for count variables; and student’s t distributions were used for continuous variables. Parameter estimates (maximum a posterior probability (MAP) estimates) were extracted along with 95% credible intervals (CIs) and variance explained (R^2^) values for each model. We used 95% CIs to determine whether the R^2^ values were meaningful (i.e., the interval did not contain zero). Furthermore, for a traditional cut-off of α = 0.05, a power analysis indicated that we had 80% power to detect an odds ratio of 1.0693 per one standard deviation increase in a predictor variable^32^.

## Results

### Childhood temperament predicting adult outcomes

**Figure 1:**
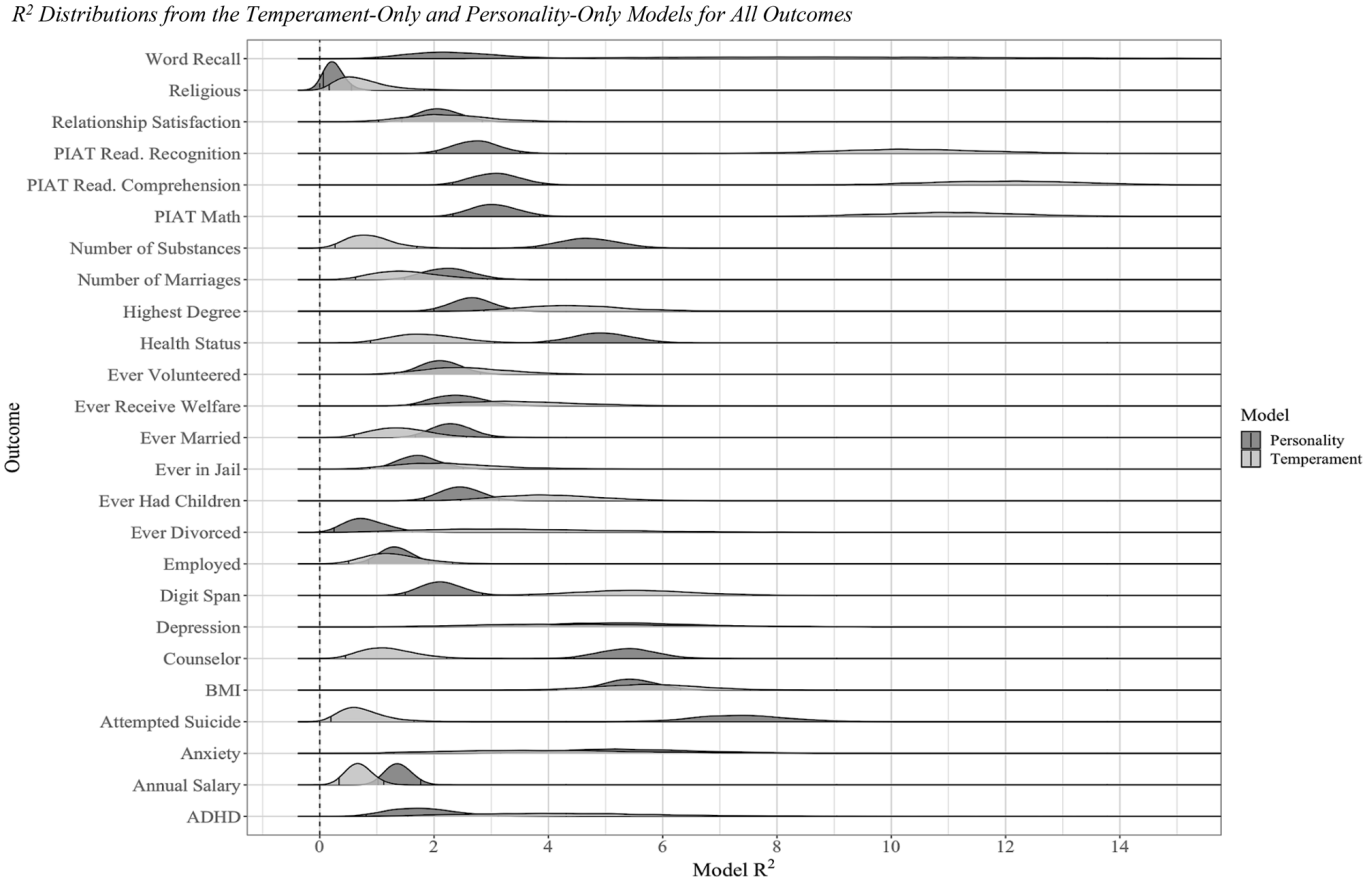
R^2^ distributions from the temperament-only and personality-only models for all outcomes. R^2^ distributions for temperament- and personality-only models are presented above for all outcomes. R^2^ values are presented as percentages. The R^2^ for the temperament-only models is plotted in light gray. The R^2^ for the adult-based personality-only models is plotted in dark gray. The 95% credible intervals, representing the R^2^ values that were present in 95% of the posterior distributions, are outlined in each distribution.

Generally, childhood personality was a good predictor of future life outcomes, up to 30 years later (Table 1). For example, temperament was related to objective indicators such as BMI (5.76%), educational attainment (4.44%), and being incarcerated (2.25%) over 2 decades later. Temperament was not associated with every outcome, however, even for outcomes that personality traits predicted (e.g., annual salary (0.69%)). Educational attainment (4.44% vs 2.67%) and substance use (0.87% vs 4.71%), as two examples, demonstrate the difference in predictive validity for childhood and adulthood personality, respectively. Overall, despite being much closer in years between assessment and outcome, the explained variance from adult personality models was not that much greater than that of childhood temperament (Fig. 1).

**Table 1:** Incremental and model R^2^ values from temperament-only, personality-only, and combined models for all outcomes.

Domain	Outcome	Model type						
		Temperament		Personality		Combined		Incremental
		*R*^*2*^	*CI*	*R*^*2*^	*CI*	*R*^*2*^	*CI*	*R*^*2*^
Health	Health status at last wave	1.86	[0.89, 3.06]	**4.95**	[4.01, 5.93]	7.67	[5.58, 9.91]	2.72^P^
	BMI at last wave	**5.76**	[4.14, 7.53]	5.44	[4.57, 6.31]	5.92	[4.30, 7.76]	0.48
Internalizing	Anxiety	4.16	[1.29, 8.09]	**5.42**	[3.17, 8.02]	9.43	[4.85, 14.57]	4.01
	Depression	4.85	[1.63, 9.13]	**5.30**	[3.00, 7.94]	15.16	[8.97, 21.21]	**9.86**^** T**^
	Counselor	1.21	[0.45, 2.22]	**5.39**	[4.45, 6.39]	7.90	[5.86, 9.97]	2.51^P^
	Suicide	0.74	[0.19, 1.65]	**7.37**	[5.90, 8.88]	9.14	[6.28, 12.29]	1.77^P^
Externalizing	ADHD	**4.35**	[1.54, 8.20]	1.82	[0.81, 3.12]	7.29	[3.53, 11.96]	**5.47**^** T**^
	Ever jail	**2.25**	[0.88, 4.21]	1.75	[1.12, 2.51]	4.45	[2.36, 7.18]	2.70
	Number of substances	0.87	[0.27, 1.70]	**4.71**	[3.78, 5.71]	6.82	[4.76, 9.06]	2.11^P^
Cognitive	Digit span	**5.54**	[3.66, 7.58]	2.13	[1.50, 2.85]	7.56	[5.39, 9.90]	**5.42**^** T**^
	Word recall	**9.01**	[3.45, 15.67]	2.37	[1.02, 4.07]	11.24	[5.01, 18.38]	**8.88**^** T**^
	PIAT math	**11.04**	[8.56, 13.60]	3.06	[2.33, 3.85]	13.66	[10.96, 16.41]	**10.60**^** T**^
	PIAT read comprehension	**12.01**	[9.45, 14.61]	3.08	[2.32, 3.90]	13.80	[11.20, 16.47]	**10.72**^** T**^
	PIAT read recognition	**10.36**	[7.95, 12.85]	2.74	[2.04, 3.50]	11.99	[9.63, 14.52]	**9.25**^** T**^
Relationship & family	Ever married	1.44	[0.60, 2.57]	**2.31**	[1.67, 3.01]	4.02	[2.51, 5.79]	1.71
	Ever divorced	**3.57**	[1.02, 7.29]	0.82	[0.25, 1.62]	5.66	[2.24, 10.03]	**4.84**^** T**^
	Times married	1.57	[0.62, 2.93]	**2.26**	[1.48, 3.10]	4.28	[2.40, 6.76]	2.03
	Relationship satisfaction	**2.25**	[1.03, 3.73]	2.10	[1.43, 2.84]	4.57	[2.84, 6.57]	2.47
	Ever have children	**4.01**	[2.46, 5.76]	2.47	[1.82, 3.14]	5.42	[3.64, 7.32]	**2.95**^** T**^
Education, career, financial	Highest degree	**4.44**	[2.87, 6.29]	2.67	[2.00, 3.39]	7.87	[5.82, 10.06]	**5.20**^** T**^
	Employed at last wave	1.30	[0.50, 2.32]	**1.34**	[0.85, 1.90]	2.39	[1.21, 3.82]	1.04
	Annual salary	0.69	[0.34, 1.12]	**1.37**	[1.02, 1.77]	1.29	[0.80, 1.90]	− 0.09
	Ever receive welfare	**3.46**	[1.59, 5.84]	2.43	[1.66, 3.36]	6.60	[3.72, 10.22]	**4.16**^** T**^
Civic engagement	Religious	**0.74**	[0.16, 1.82]	0.24	[0.06, 0.55]	2.33	[0.78, 4.95]	**2.08**^** T**^
	Ever volunteered	**2.58**	[1.30, 4.15]	2.12	[1.48, 2.81]	4.53	[2.85, 6.54]	**2.41**^** T**^

R^2^ values are presented as percentages. Results are from models without covariates. The incremental model R^2^ is the percentage of the combined model R^2^ not accounted for by adult-based personality (i.e., has personality model R^2^ subtracted out). Bold values indicate the larger R^2^ value between the temperament-only and personality-only models for each outcome. In the “Incremental” column, if the combined and personality-only R^2^ credible intervals did not overlap, then temperament was a meaningful predictor above and beyond personality in terms of incremental predictive validity (these values are marked by a T superscript). Alternatively, if the combined and temperament-only R^2^ credible intervals did not overlap, then personality was a meaningful predictor above and beyond temperament (these values are marked by a P superscript). To ease in readability, values in the “Incremental” column indicate outcomes for which temperament explained incremental variance are also bolded.

Since the primary goal of this paper was to view the sole and incremental explanatory power of the temperament relative to personality (i.e., the model R^2^ values), individual temperament estimates with the outcomes were of lesser interest. However, these estimates can be found in Supplementary Tables S1–S7 from models without covariates; Supplementary Tables S8a–S14a from models with covariates; and Supplementary Table S15 for a comparison of estimates from models with and without covariates.

### Independent associations of childhood and adulthood personality for life outcomes

Next, we sought to examine whether childhood temperament yielded incremental predictive validity of life outcomes over adult personality. For these models, all childhood temperament characteristics and all adult-based personality traits were entered as predictors simultaneously. To determine the incremental R^2^ value for childhood temperament within the combined model for any given outcome, the R^2^ from the adult-based personality-only model was subtracted from the total R^2^ for the combined model for that outcome (Table 1; Supplementary Fig. 1). Individual estimates for each outcome from the personality-only models can be found in Supplementary Tables S1–S7 (without covariates); Supplementary Tables S8b–S14b (with covariates); and Supplementary Table S15 for a comparison of estimates from models with and without covariates.

In general, temperament provided a number of incremental predictions above personality, despite personality being assessed closer in time, as temperament was, on average, assessed over 20 years prior to these outcomes (Table 1). Cognitive outcomes, a diagnosis of depression or ADHD, and highest degree obtained were amongst the most prominent outcomes in which temperament provided incremental variance above adult-based personality. Incremental variances explained for temperament ranged from just above 2% to above 10%—levels of association that are high for psychology, especially when considering the nearly 30-year timespan. For individual estimates for each outcome from the combined models, see Supplementary Tables S1–S7 (without covariates) and Supplementary Tables S8c–S14c (with covariates).

## Discussion

Within this paper, we tested the predictive validity of childhood personality for life outcomes up to 30 years later. Two main findings emerged. First, temperament measured between ages 0–6 was able to predict a wide-ranging number of life outcomes. Second, temperament often provided incremental predictive validity above adult-based personality, suggesting that there is unique information in childhood assessments despite being assessed farther away in time. These findings establish the importance of both distal and proximal personality predictors of outcomes, supporting the need to understand who an individual is throughout the lifespan.

### Predictive validity of temperament

For a set of traits that were measured between infancy and age six, the ability of temperament to predict outcomes in adulthood, decades later, was noteworthy. Consistent with past research^33,34^, our temperament assessments completed at an average age of 3.76 years lend support that personality can be measured early on in life and have predictive validity for important life outcomes decades later.

Our wide-ranging array of outcome variables further supports the broad and far-reaching predictive abilities of childhood temperament. Many past studies with childhood and adulthood personality often limited their investigations of prediction with temperament to psychopathology-related outcomes^35^ while those that examined other outcomes typically remained in a single outcome domain (e.g., occupations^36^). Thus, our study indeed found that early assessments of temperament are associated with a broad array of outcomes, up to decades later. Notable examples include BMI, cognitive ability, divorce, educational attainment, and civic engagement.

While not reported in the results but available in the supplementary materials, across all domains, the temperament trait of compliance emerged as the most frequent individual predictor, followed closely by sociability and predictability. Compliance is believed to represent a childhood precursor of agreeableness, but agreeableness-related traits are typically not included as a major dimension in popular temperament models but are included in childhood personality models inspired by the Big Five^33^. Part of this could be due to variation in methodology of assessing these temperament traits, as this agreeableness-related factor is the broadest and largest dimension that has emerged from parental descriptions of child temperament^37^ but emerges less frequently through other assessment methods (e.g., self-report, laboratory tasks). Since this trait was in fact reported on by parents in our study, its prominence in predicting outcomes could reflect the parents’ concern with managing the child’s behavior and avoiding parent–child conflict, thus perhaps over-reporting on or emphasizing this quality in their child. Agreeableness as a personality trait is related to outcomes in various domains, including interpersonal, social, and health outcomes^11,38–40^ so it is not entirely surprising this possible childhood precursor of agreeableness is related to a vast number of outcomes as well. Furthermore, one empirically derived personality taxonomy for children, the Hierarchical Personality Inventory for Children (HiPIC^41^), found that compliance represented a blend of benevolence and, more interestingly, conscientiousness. Given conscientiousness’s many associations with beneficial outcomes^39,42–44^, our findings of compliance being associated with the greatest number of outcomes is perhaps even more to be expected.

There are also reasons as to why the other two most frequent temperament predictors, predictability and sociability, emerged as often as they did. First, predictability, also sometimes called regularity, refers to the “predictability” of a child’s biological and behavioral patterns^45,46^. With age, the children’s daily schedules and personal habits also appear to be consistent with their earlier predictability levels. Highly regular children like setting schedules for accomplishing tasks and enjoy structure in their lives, whereas highly irregular children have more difficulty adapting to set routines and forming regular habits and mood patterns, which can precede behavioral problems later in life^47–49^. However, children exhibiting these irregular tendencies also can adjust better to unexpected changes in their routine and are more flexible in lifestyle changes. Predictability’s associations with setting schedules and routines as well as consistent mood patterns is reflective of both conscientiousness and neuroticism; two traits that are associated with numerous outcomes in many domains^39,50^.

Additionally, past research has suggested sociability be considered a lower-order quality of the broader dimension positive emotionality as opposed to constituting its own independent trait^37^. Positive emotionality and the qualities it is believed to subsume (e.g., sociability, shyness, dominance) are related to future scores on extraversion^5,51^. Extraversion is linked to positive outcomes in adulthood, particularly those related to social and well-being outcomes^52–55^. Greater well-being itself is positively related to beneficial outcomes in several life domains^39^, serving as one path by which childhood sociability is linked to outcomes in different domains.

### Reasons for incremental predictive validity of temperament over personality

We found evidence for a lifespan perspective, such that it mattered “where you start and finish.” It is beneficial to measure individual differences more than a single time over the life course, with childhood being an important time period for understanding adult outcomes. Not only did temperament provide incremental validity, but it evidenced stronger initial predictions across a number of outcomes, despite the fact that the lag in time between assessment and outcome was decades longer for temperament than personality.

A few reasons may explain why childhood is important to understand adult outcomes. First, these results suggest there are childhood-specific processes, as outlined by Hill et al.^13^, that relate childhood individual differences to adult outcomes that are separate from adult processes (i.e., differential pathways). For example, personality measures better predicted substance use compared to temperament assessments. One potential reason why is that the processes that relate individual differences to those outcomes are more relevant for adults than children. Behaviors that influence substance use are better assessed with adult personality measures because they either have content that better assesses those process or because the processes are assessed closer in time to outcomes. This reasoning could similarly be why temperament better predicted an ADHD diagnosis, as diagnoses are often made around age six^56^.

Alternatively, the opportunities and snares hypothesis could offer another explanation. As Hill et al.^13^ point out, childhood personality measures are important because of the sensitive period of childhood due to its time-limited nature. The development that occurs early in life can be consequential to future outcomes, especially if this development primarily occurs in a limited span of time and/or the paths one is then led down cannot be reversed. If temperament traits are an acceptable proxy of an infant or child’s functioning and healthy development, when cognitive abilities are also being largely formed and solidified (especially apparent when considering the long-term stability of IQ^57^), then future personality traits would offer little, if any, predictive validity not already captured by temperament. This could explain why temperament assessments did a good job at predicting educational outcomes over and above personality because education is an important childhood experience that is cumulative in nature.

## Limitations and conclusion

While our study was a powerful test of the incremental predictive validity of temperament compared to the Big Five personality traits using a representative sample assessed over 3 decades, there were a few limitations. First, we were limited by our measures. The temperament traits we could examine was restricted to what was included in the survey, and thus we could not include some regularly examined qualities such as effortful control or behavioral inhibition^58,59^. For adulthood personality, the most data were available for the TIPI, which is a relatively brief measure. However, when examining if the amount of variance explained by adult personality was similar when using a more comprehensive measure (i.e., the mini-IPIP^50^), albeit with a smaller sample size, the values were very similar (Table S17; see also Table S18 for a comparison of the estimates with the TIPI versus mini-IPIP). This suggests that the TIPI captured an acceptable amount of variance to be explained in our outcomes by the Big Five traits. Ideally, to best test the question of if there is incremental validity of childhood personality compared to adulthood personality, comprehensive measures of both sets of traits are needed. Thus, our study should be considered a first step in examining this and future work is needed to confirm and expand upon the results. Second, different reporting methods were used (parent, self) which have been differently associated with life outcomes^42,60^. Third, an alternative explanation for the findings is content and/or structural differences between the two sets of individual differences^2,61^. It is hard to address whether these factors are driving the differences as it is difficult to take an adult taxonomy and apply it to children. Behavioral expression of personality differs across age which is one of the reasons why the Little Six^2^ rather than the Big Five, for example, is found in childhood.

Using a large-scale longitudinal study across a 30-year time frame, we identified non-redundant predictions of life outcomes for temperament and personality. Temperament explained the most variance for outcomes such as cognitive ability and educational attainment whereas personality performed best for outcomes such as health status, substance use, and most internalizing outcomes. Our results highlight the benefit of a lifespan approach to understanding life outcomes, where adult-based outcomes are informed by child-based assessments.

## Supplementary Information

Supplementary Information.

## Data Availability

Data are drawn from the publicly available National Longitudinal Survey of Youth 1979—Child and Young Adult sample^22^ which is freely accessible at https://www.nlsinfo.org/investigator. The raw data used for the current study are available at the study’s OSF page (https://osf.io/kyrq7/).
