# Supplementary material for: Childhood temperament and adulthood personality differentially predict life outcomes
Source: Sci Rep. 2022 Jun 18;12:10286. doi: 10.1038/s41598-022-14666-0 (PMC9206675; doi:10.1038/s41598-022-14666-0)
Supplement: Supplementary file 1 — Supplementary Information. [file 41598_2022_14666_MOESM1_ESM.docx]

Supplementary materials for:

**Childhood temperament and adulthood personality differentially predict life outcomes**

Amanda J. Wright^*^

Joshua J. Jackson

| **Table S1** | | | | | |
| --- | --- | --- | --- | --- | --- |
| *Individual Estimates from the Childhood Temperament-Only, Adult-Based Personality Only, and Combined Models Without Covariates for Health Outcomes* | | | | | |
|  |  | Health Status | | BMI | |
| Model | | *b* | *CI* | *b* | *CI* |
| Temperament | | | | | |
|  | Activity | **-0.09** | **[-0.18, -0.01]** | 0.03 | [-0.01, 0.06] |
|  | Compliance | **0.13** | **[0.04, 0.21]** | **-0.06** | **[-0.10, -0.02]** |
|  | Fearful | -0.03 | [-0.13, 0.06] | 0.00 | [-0.05, 0.04] |
|  | Insecure | **-0.12** | **[-0.21, -0.03]** | 0.00 | [-0.04, 0.04] |
|  | Pos. Affect | 0.01 | [-0.06, 0.09] | 0.02 | [-0.01, 0.06] |
|  | Predictability | -0.02 | [-0.11, 0.07] | -0.04 | [-0.08, 0.00] |
|  | Sociability | 0.05 | [-0.04, 0.14] | 0.00 | [-0.04, 0.03] |
| Personality | | | | | |
|  | E | **0.15** | **[0.11, 0.19]** | 0.00 | [-0.02, 0.02] |
|  | A | 0.00 | [-0.05, 0.04] | -0.02 | [-0.04, 0.00] |
|  | C | **0.16** | **[0.11, 0.21]** | 0.00 | [-0.02, 0.03] |
|  | N | **-0.28** | **[-0.32, -0.23]** | 0.01 | [-0.01, 0.03] |
|  | O | 0.02 | [-0.03, 0.07] | 0.00 | [-0.02, 0.02] |
| Combined | | | | | |
|  | Activity | **-0.10** | **[-0.18, -0.01]** | 0.03 | [-0.01, 0.07] |
|  | Compliance | **0.11** | **[0.02, 0.20]** | **-0.07** | **[-0.11, -0.03]** |
|  | Fearful | -0.02 | [-0.12, 0.07] | 0.00 | [-0.05, 0.04] |
|  | Insecure | **-0.13** | **[-0.22, -0.03]** | 0.00 | [-0.04, 0.04] |
|  | Pos. Affect | 0.00 | [-0.08, 0.08] | 0.03 | [-0.01, 0.06] |
|  | Predictability | -0.03 | [-0.12, 0.06] | -0.04 | [-0.08, -0.00] |
|  | Sociability | 0.04 | [-0.05, 0.13] | 0.00 | [-0.04, 0.04] |
|  | E | **0.18** | **[0.09, 0.27]** | 0.04 | [0.00, 0.08] |
|  | A | -0.03 | [-0.13, 0.06] | 0.01 | [-0.03, 0.05] |
|  | C | **0.26** | **[0.17, 0.35]** | 0.01 | [-0.03, 0.05] |
|  | N | **-0.26** | **[-0.35, -0.17]** | **0.07** | **[0.03, 0.11]** |
|  | O | -0.09 | [-0.19, 0.00] | 0.01 | [-0.03, 0.05] |
| *Note*. *b* = regression coefficient. *CI* = 95% credible interval. E = extraversion. A = agreeableness. C = conscientiousness. N = neuroticism. O = openness. Pos. Affect = positive affect. Bold values indicate that the credible intervals do not contain 0.00. Results are from models without covariates. All traits are standardized. | | | | | |

| **Table S2** | | | | | | | | | |
| --- | --- | --- | --- | --- | --- | --- | --- | --- | --- |
| *Individual Estimates from the Childhood Temperament-Only, Adult-Based Personality Only, and Combined Models Without Covariates for Internalizing Outcomes* | | | | | | | | | |
|  |  | Anxiety | | Depression | | Counselor | | Suicide | |
| Model | | *OR* | *CI* | *OR* | *CI* | *OR* | *CI* | *OR* | *CI* |
| Temperament | | | | | | | | | |
|  | Activity | **1.32** | **[1.03, 1.70]** | 1.20 | [0.91, 1.58] | 1.00 | [0.91, 1.10] | 0.93 | [0.80, 1.07] |
|  | Compliance | 1.01 | [0.78, 1.32] | 0.98 | [0.74, 1.30] | **0.83** | **[0.74, 0.91]** | 0.95 | [0.81, 1.11] |
|  | Fearful | 1.04 | [0.77, 1.37] | 0.88 | [0.64, 1.21] | 0.99 | [0.89, 1.11] | 1.01 | [0.84, 1.20] |
|  | Insecure | 0.92 | [0.69, 1.21] | 1.33 | [1.00, 1.78] | 1.04 | [0.94, 1.15] | 1.08 | [0.92, 1.27] |
|  | Pos. Affect | 1.01 | [0.79, 1.31] | 0.96 | [0.75, 1.25] | 1.00 | [0.92, 1.09] | 1.01 | [0.89, 1.14] |
|  | Predictability | 1.25 | [0.92, 1.68] | 1.01 | [0.73, 1.38] | 1.06 | [0.96, 1.18] | 1.06 | [0.91, 1.24] |
|  | Sociability | 1.15 | [0.89, 1.49] | **1.46** | **[1.10, 1.93]** | 1.01 | [0.92, 1.12] | 1.15 | [0.98, 1.36] |
| Personality | | | | | | | | | |
|  | E | 0.88 | [0.76, 1.00] | **0.80** | **[0.70, 0.92]** | 1.04 | [0.99, 1.09] | **0.87** | **[0.81, 0.95]** |
|  | A | **1.24** | **[1.08, 1.44]** | **1.19** | **[1.03, 1.38]** | **1.09** | **[1.04, 1.15]** | **1.19** | **[1.09, 1.30]** |
|  | C | **1.15** | **[1.01, 1.32]** | 1.05 | [0.92, 1.21] | **0.83** | **[0.79, 0.87]** | **0.81** | **[0.75, 0.87]** |
|  | N | **1.63** | **[1.42, 1.88]** | **1.58** | **[1.38, 1.81]** | **1.54** | **[1.46, 1.63]** | **2.00** | **[1.84, 2.17]** |
|  | O | **1.21** | **[1.04, 1.40]** | **1.23** | **[1.06, 1.44]** | **1.14** | **[1.09, 1.21]** | **1.37** | **[1.26, 1.50]** |
| Combined | | | | | | | | | |
|  | Activity | **1.32** | **[1.01, 1.74]** | 1.17 | [0.86, 1.58] | 0.98 | [0.89, 1.09] | 0.89 | [0.76, 1.03] |
|  | Compliance | 1.01 | [0.76, 1.34] | 0.91 | [0.67, 1.23] | **0.82** | **[0.74, 0.92]** | 0.96 | [0.82, 1.13] |
|  | Fearful | 1.03 | [0.76, 1.41] | 0.84 | [0.59, 1.18] | 0.98 | [0.87, 1.09] | 0.98 | [0.82, 1.16] |
|  | Insecure | 0.87 | [0.66, 1.15] | 1.23 | [0.91, 1.67] | 1.00 | [0.89, 1.11] | 1.00 | [0.84, 1.18] |
|  | Pos. Affect | 1.04 | [0.79, 1.36] | 1.03 | [0.78, 1.38] | 1.01 | [0.92, 1.11] | 1.04 | [0.90, 1.20] |
|  | Predictability | 1.22 | [0.88, 1.71] | 1.05 | [0.74, 1.50] | 1.08 | [0.97, 1.19] | 1.11 | [0.94, 1.31] |
|  | Sociability | 1.09 | [0.83, 1.46] | **1.48** | **[1.09, 2.05]** | 1.00 | [0.89, 1.11] | 1.16 | [0.98, 1.37] |
|  | E | 1.12 | [0.84, 1.48] | **0.65** | **[0.47, 0.90]** | 1.00 | [0.91, 1.11] | **0.76** | **[0.65, 0.89]** |
|  | A | **1.36** | **[1.01, 1.85]** | **1.45** | **[1.06, 2.01]** | 1.06 | [0.96, 1.18] | 1.07 | [0.91, 1.27] |
|  | C | 1.14 | [0.87, 1.49] | 1.20 | [0.91, 1.61] | **0.86** | **[0.78, 0.95]** | 0.92 | [0.79, 1.07] |
|  | N | **1.56** | **[1.23, 2.01]** | **1.82** | **[1.39, 2.40]** | **1.68** | **[1.50, 1.88]** | **2.07** | **[1.78, 2.42]** |
|  | O | 1.04 | [0.78, 1.39] | 1.38 | [1.00, 1.90] | **1.20** | **[1.08, 1.33]** | **1.30** | **[1.10, 1.53]** |
| *Note*. *OR* = odds ratio. *CI* = 95% credible interval. E = extraversion. A = agreeableness. C = conscientiousness. N = neuroticism. O = openness. Pos. Affect = positive affect. Bold values indicate that the credible intervals do not contain 1.00. Results are from models without covariates. All traits are standardized. | | | | | | | | | |

| **Table S3** | | | | | | | |
| --- | --- | --- | --- | --- | --- | --- | --- |
| *Individual Estimates from the Childhood Temperament-Only, Adult-Based Personality Only, and Combined Models Without Covariates for Externalizing Outcomes* | | | | | | | |
|  |  | ADHD | | Ever Been in Jail | | Number of Substances | |
| Model | | *OR* | *CI* | *OR* | *CI* | *IRR* | *CI* |
| Temperament | | | | | | | |
|  | Activity | 1.02 | [0.78, 1.32] | 1.00 | [0.82, 1.21] | 0.99 | [0.96, 1.02] |
|  | Compliance | **0.67** | **[0.51, 0.86]** | 1.00 | [0.83, 1.22] | 0.99 | [0.96, 1.03] |
|  | Fearful | 0.87 | [0.64, 1.15] | 1.15 | [0.94, 1.39] | 1.01 | [0.98, 1.05] |
|  | Insecure | 0.78 | [0.59, 1.05] | 1.12 | [0.93, 1.37] | **0.96** | **[0.93, 0.99]** |
|  | Pos. Affect | 0.90 | [0.73, 1.12] | 1.08 | [0.92, 1.28] | 0.99 | [0.96, 1.01] |
|  | Predictability | 1.13 | [0.86, 1.49] | **0.72** | **[0.61, 0.86]** | 1.01 | [0.98, 1.04] |
|  | Sociability | 0.81 | [0.63, 1.05] | **0.77** | **[0.63, 0.94]** | 1.01 | [0.98, 1.04] |
| Personality | | | | | | | |
|  | E | 1.10 | [0.96, 1.27] | **0.77** | **[0.71, 0.84]** | **1.04** | **[1.03, 1.06]** |
|  | A | 0.94 | [0.82, 1.07] | **0.75** | **[0.69, 0.81]** | **0.97** | **[0.95, 0.98]** |
|  | C | **0.72** | **[0.64, 0.82]** | 1.07 | [0.99, 1.17] | **0.94** | **[0.92, 0.95]** |
|  | N | 1.08 | [0.94, 1.23] | **1.16** | **[1.06, 1.26]** | **1.07** | **[1.06, 1.09]** |
|  | O | 0.98 | [0.86, 1.12] | **1.20** | **[1.10, 1.31]** | **1.07** | **[1.05, 1.09]** |
| Combined | | | | | | | |
|  | Activity | 0.98 | [0.75, 1.26] | 0.99 | [0.82, 1.19] | 0.99 | [0.96, 1.02] |
|  | Compliance | **0.65** | **[0.50, 0.85]** | 1.02 | [0.83, 1.24] | 1.00 | [0.97, 1.03] |
|  | Fearful | 0.88 | [0.65, 1.18] | 1.14 | [0.94, 1.39] | 1.02 | [0.98, 1.05] |
|  | Insecure | 0.77 | [0.56, 1.03] | 1.11 | [0.91, 1.36] | **0.95** | **[0.92, 0.98]** |
|  | Pos. Affect | 0.90 | [0.72, 1.13] | 1.07 | [0.90, 1.26] | 0.99 | [0.96, 1.01] |
|  | Predictability | 1.10 | [0.84, 1.48] | **0.75** | **[0.63, 0.90]** | 1.00 | [0.98, 1.04] |
|  | Sociability | 0.77 | [0.58, 1.02] | **0.78** | **[0.64, 0.96]** | 1.00 | [0.97, 1.03] |
|  | E | 1.31 | [0.99, 1.75] | **0.74** | **[0.61, 0.90]** | **1.05** | **[1.02, 1.08]** |
|  | A | 0.89 | [0.69, 1.17] | **0.69** | **[0.57, 0.84]** | **0.95** | **[0.93, 0.98]** |
|  | C | 0.78 | [0.59, 1.02] | 0.87 | [0.72, 1.06] | **0.92** | **[0.90, 0.95]** |
|  | N | 1.19 | [0.92, 1.55] | 1.01 | [0.83, 1.23] | **1.08** | **[1.05, 1.12]** |
|  | O | 0.91 | [0.68, 1.22] | **1.31** | **[1.05, 1.63]** | **1.08** | **[1.04, 1.11]** |
| *Note*. *OR* = odds ratio. *IRR* = incidence rate ratio. *CI* = 95% credible interval. E = extraversion. A = agreeableness. C = conscientiousness. N = neuroticism. O = openness. Pos. Affect = positive affect. Bold values indicate that the credible intervals do not contain 1.00. Results are from models without covariates. All traits are standardized. | | | | | | | |

| **Table S4** | | | | | | | | | | | |
| --- | --- | --- | --- | --- | --- | --- | --- | --- | --- | --- | --- |
| *Individual Estimates from the Childhood Temperament-Only, Adult-Based Personality Only, and Combined Models Without Covariates for Cognitive Outcomes* | | | | | | | | | | | |
|  |  | Digit Span | | Word Recall | | PIAT Math | | PIAT Read Comp | | PIAT Read Rec | |
| Model | | *b* | *CI* | *b* | *CI* | *b* | *CI* | *b* | *CI* | *b* | *CI* |
| Temperament | | | | | | | | | | | |
|  | Activity | **-0.08** | **[-0.13, -0.04]** | **-0.16** | **[-0.31, -0.01]** | **-0.09** | **[-0.13, -0.04]** | **-0.05** | **[-0.10, -0.01]** | **-0.07** | **[-0.12, -0.02]** |
|  | Compliance | **0.10** | **[0.05, 0.15]** | 0.01 | [-0.14, 0.15] | **0.12** | **[0.07, 0.17]** | **0.14** | **[0.09, 0.18]** | **0.12** | **[0.07, 0.17]** |
|  | Fearful | 0.02 | [-0.04, 0.07] | -0.11 | [-0.26, 0.05] | **-0.09** | **[-0.14, -0.04]** | **-0.08** | **[-0.13, -0.03]** | **-0.07** | **[-0.12, -0.02]** |
|  | Insecure | -0.04 | [-0.09, 0.01] | -0.11 | [-0.27, 0.04] | **-0.07** | **[-0.12, -0.02]** | -0.05 | [-0.10, -0.00] | -0.04 | [-0.09, 0.01] |
|  | Pos. Affect | 0.00 | [-0.04, 0.05] | 0.10 | [-0.03, 0.23] | -0.02 | [-0.06, 0.02] | **-0.05** | **[-0.09, -0.01]** | **-0.05** | **[-0.09, -0.01]** |
|  | Predictability | 0.01 | [-0.04, 0.06] | 0.09 | [-0.06, 0.24] | **0.09** | **[0.04, 0.14]** | **0.09** | **[0.05, 0.14]** | **0.10** | **[0.05, 0.15]** |
|  | Sociability | **0.17** | **[0.12, 0.22]** | 0.10 | [-0.05, 0.25] | **0.24** | **[0.18, 0.29]** | **0.25** | **[0.20, 0.29]** | **0.23** | **[0.18, 0.28]** |
| Personality | | | | | | | | | | | |
|  | E | **0.10** | **[0.08, 0.12]** | **0.09** | **[0.04, 0.15]** | **0.10** | **[0.07, 0.12]** | **0.09** | **[0.07, 0.12]** | **0.08** | **[0.06, 0.11]** |
|  | A | **0.03** | **[0.01, 0.06]** | **0.08** | **[0.02, 0.13]** | 0.01 | [-0.01, 0.03] | **0.08** | **[0.05, 0.10]** | **0.07** | **[0.05, 0.10]** |
|  | C | **-0.05** | **[-0.07, -0.02]** | 0.01 | [-0.05, 0.07] | **-0.13** | **[-0.16, -0.11]** | **-0.11** | **[-0.14, -0.09]** | **-0.09** | **[-0.11, -0.06]** |
|  | N | -0.02 | [-0.05, 0.00] | -0.03 | [-0.09, 0.03] | **-0.09** | **[-0.12, -0.07]** | **-0.06** | **[-0.08, -0.03]** | **-0.05** | **[-0.07, -0.02]** |
|  | O | **0.07** | **[0.04, 0.09]** | 0.01 | [-0.05, 0.07] | **0.05** | **[0.02, 0.08]** | **0.06** | **[0.03, 0.08]** | **0.07** | **[0.04, 0.10]** |
| Combined | | | | | | | | | | | |
|  | Activity | **-0.08** | **[-0.12, -0.03]** | -0.15 | [-0.31, -0.00] | **-0.08** | **[-0.13, -0.03]** | -0.05 | [-0.09, -0.00] | **-0.06** | **[-0.11, -0.02]** |
|  | Compliance | **0.10** | **[0.05, 0.15]** | 0.01 | [-0.14, 0.16] | **0.12** | **[0.07, 0.17]** | **0.13** | **[0.09, 0.18]** | **0.12** | **[0.07, 0.16]** |
|  | Fearful | 0.02 | [-0.03, 0.08] | -0.11 | [-0.28, 0.05] | **-0.08** | **[-0.13, -0.03]** | **-0.07** | **[-0.12, -0.03]** | **-0.07** | **[-0.12, -0.01]** |
|  | Insecure | -0.04 | [-0.09, 0.01] | -0.10 | [-0.26, 0.05] | -0.05 | [-0.10, -0.00] | -0.04 | [-0.08, 0.01] | -0.03 | [-0.08, 0.02] |
|  | Pos. Affect | 0.00 | [-0.04, 0.04] | 0.10 | [-0.03, 0.23] | -0.02 | [-0.06, 0.02] | **-0.05** | **[-0.09, -0.01]** | **-0.05** | **[-0.09, -0.01]** |
|  | Predictability | 0.01 | [-0.04, 0.05] | 0.09 | [-0.06, 0.24] | **0.08** | **[0.03, 0.12]** | **0.08** | **[0.04, 0.13]** | **0.09** | **[0.04, 0.14]** |
|  | Sociability | **0.16** | **[0.11, 0.21]** | 0.10 | [-0.05, 0.25] | **0.23** | **[0.18, 0.28]** | **0.24** | **[0.19, 0.29]** | **0.22** | **[0.17, 0.27]** |
|  | E | **0.08** | **[0.03, 0.12]** | -0.02 | [-0.17, 0.13] | 0.04 | [-0.01, 0.09] | 0.02 | [-0.02, 0.07] | 0.01 | [-0.04, 0.06] |
|  | A | -0.03 | [-0.08, 0.02] | 0.05 | [-0.10, 0.21] | -0.02 | [-0.07, 0.03] | **0.07** | **[0.02, 0.11]** | **0.07** | **[0.02, 0.12]** |
|  | C | -0.03 | [-0.08, 0.03] | 0.00 | [-0.17, 0.16] | **-0.13** | **[-0.18, -0.08]** | **-0.10** | **[-0.15, -0.05]** | **-0.08** | **[-0.12, -0.03]** |
|  | N | -0.02 | [-0.07, 0.03] | -0.03 | [-0.19, 0.12] | **-0.13** | **[-0.18, -0.08]** | **-0.06** | **[-0.11, -0.01]** | **-0.05** | **[-0.10, -0.01]** |
|  | O | **0.09** | **[0.05, 0.14]** | 0.01 | [-0.16, 0.18] | **0.06** | **[0.01, 0.11]** | **0.05** | **[0.01, 0.10]** | **0.06** | **[0.01, 0.11]** |
| *Note*. *b* = regression coefficient. *CI* = 95% credible interval. E = extraversion. A = agreeableness. C = conscientiousness. N = neuroticism. O = openness. Pos. Affect = positive affect. Bold values indicate that the credible intervals do not contain 0.00. Results are from models without covariates. All traits are standardized. | | | | | | | | | | | |

| **Table S5** | | | | | | | | | | | |
| --- | --- | --- | --- | --- | --- | --- | --- | --- | --- | --- | --- |
| *Individual Estimates from the Childhood Temperament-Only, Adult-Based Personality Only, and Combined Models Without Covariates for Relationship and Family Outcomes* | | | | | | | | | | | |
|  |  | Ever Married | | Ever Divorced | | Times Married | | Relationship Satisfaction | | Ever Had Children | |
| Model | | *OR* | *CI* | *OR* | *CI* | *IRR* | *OR* | *b* | *CI* | *OR* | *CI* |
| Temperament | | | | | | | | | | | |
|  | Activity | 1.05 | [0.94, 1.18] | 1.09 | [0.80, 1.50] | 1.04 | [0.94, 1.15] | -0.03 | [-0.14, 0.08] | **1.18** | **[1.06, 1.31]** |
|  | Compliance | **1.23** | **[1.09, 1.40]** | 0.74 | [0.54, 1.00] | **1.17** | **[1.05, 1.31]** | **0.21** | **[0.09, 0.33]** | 1.05 | [0.94, 1.17] |
|  | Fearful | 0.92 | [0.81, 1.06] | 0.80 | [0.53, 1.18] | 0.93 | [0.83, 1.05] | -0.06 | [-0.18, 0.07] | 1.12 | [0.99, 1.24] |
|  | Insecure | 0.99 | [0.87, 1.13] | 0.88 | [0.63, 1.21] | 0.99 | [0.89, 1.10] | -0.10 | [-0.21, 0.01] | **1.21** | **[1.08, 1.35]** |
|  | Pos. Affect | 0.93 | [0.84, 1.02] | 0.90 | [0.73, 1.13] | 0.93 | [0.86, 1.01] | 0.03 | [-0.08, 0.12] | 0.97 | [0.89, 1.07] |
|  | Predictability | 1.01 | [0.90, 1.14] | 1.04 | [0.76, 1.41] | 1.01 | [0.91, 1.13] | 0.06 | [-0.05, 0.17] | **0.78** | **[0.70, 0.86]** |
|  | Sociability | 1.09 | [0.97, 1.23] | 0.91 | [0.67, 1.24] | 1.07 | [0.96, 1.19] | 0.12 | [0.00, 0.23] | **0.84** | **[0.75, 0.94]** |
| Personality | | | | | | | | | | | |
|  | E | **1.12** | **[1.06, 1.17]** | 1.03 | [0.93, 1.15] | **1.08** | **[1.03, 1.12]** | **0.13** | **[0.08, 0.19]** | 1.00 | [0.95, 1.05] |
|  | A | **1.22** | **[1.15, 1.28]** | 1.01 | [0.91, 1.12] | **1.13** | **[1.09, 1.18]** | **0.09** | **[0.03, 0.15]** | 1.05 | [1.00, 1.10] |
|  | C | **1.25** | **[1.18, 1.32]** | 1.13 | [1.00, 1.27] | **1.18** | **[1.12, 1.23]** | **0.11** | **[0.05, 0.17]** | **1.38** | **[1.31, 1.45]** |
|  | N | 1.00 | [0.95, 1.06] | **1.15** | **[1.04, 1.29]** | 1.01 | [0.96, 1.05] | **-0.19** | **[-0.25, -0.12]** | **1.14** | **[1.09, 1.21]** |
|  | O | **0.83** | **[0.78, 0.88]** | 1.11 | [1.00, 1.24] | **0.89** | **[0.85, 0.92]** | -0.02 | [-0.08, 0.04] | **0.88** | **[0.83, 0.92]** |
| Combined | | | | | | | | | | | |
|  | Activity | 1.06 | [0.94, 1.19] | 1.08 | [0.79, 1.50] | 1.05 | [0.95, 1.16] | 0.00 | [-0.12, 0.11] | **1.17** | **[1.06, 1.30]** |
|  | Compliance | **1.22** | **[1.08, 1.39]** | 0.74 | [0.54, 1.02] | **1.16** | **[1.03, 1.30]** | **0.18** | **[0.06, 0.31]** | 1.05 | [0.93, 1.17] |
|  | Fearful | 0.92 | [0.80, 1.05] | 0.81 | [0.53, 1.22] | 0.93 | [0.82, 1.04] | -0.03 | [-0.16, 0.10] | 1.11 | [0.98, 1.24] |
|  | Insecure | 0.99 | [0.86, 1.13] | 0.85 | [0.61, 1.18] | 0.99 | [0.89, 1.10] | -0.11 | [-0.23, 0.01] | **1.18** | **[1.05, 1.31]** |
|  | Pos. Affect | 0.93 | [0.84, 1.02] | 0.88 | [0.71, 1.12] | 0.93 | [0.86, 1.01] | 0.03 | [-0.07, 0.13] | 0.97 | [0.89, 1.07] |
|  | Predictability | 0.99 | [0.87, 1.13] | 1.06 | [0.79, 1.44] | 1.00 | [0.90, 1.11] | 0.05 | [-0.07, 0.17] | **0.78** | **[0.71, 0.87]** |
|  | Sociability | 1.08 | [0.95, 1.23] | 0.90 | [0.65, 1.23] | 1.06 | [0.95, 1.18] | **0.14** | **[0.01, 0.26]** | **0.85** | **[0.76, 0.94]** |
|  | E | 1.07 | [0.95, 1.21] | 1.15 | [0.84, 1.58] | 1.05 | [0.95, 1.17] | **0.16** | **[0.04, 0.27]** | 1.02 | [0.92, 1.14] |
|  | A | **1.36** | **[1.20, 1.55]** | 0.86 | [0.61, 1.21] | **1.26** | **[1.14, 1.41]** | 0.11 | [-0.01, 0.23] | 1.04 | [0.93, 1.17] |
|  | C | **1.24** | **[1.08, 1.41]** | 1.19 | [0.82, 1.74] | **1.18** | **[1.06, 1.32]** | **0.15** | **[0.04, 0.26]** | **1.26** | **[1.12, 1.40]** |
|  | N | 0.98 | [0.87, 1.11] | 1.17 | [0.83, 1.63] | 1.00 | [0.90, 1.11] | **-0.12** | **[-0.24, -0.01]** | **1.18** | **[1.06, 1.32]** |
|  | O | **0.86** | **[0.76, 0.98]** | 1.15 | [0.83, 1.63] | **0.89** | **[0.80, 0.99]** | -0.01 | [-0.14, 0.11] | **0.89** | **[0.79, 0.99]** |
| *Note*. *OR* = odds ratio. *b* = regression coefficient. *CI* = 95% credible interval. E = extraversion. A = agreeableness. C = conscientiousness. N = neuroticism. O = openness. Pos. Affect = positive affect. Bold values indicate that the credible intervals either do not contain 1.00 (for *OR*s) or do not contain 0.00 (for regression coefficients). Results are from models without covariates. | | | | | | | | | | | |

| **Table S6** | | | | | | | | | |
| --- | --- | --- | --- | --- | --- | --- | --- | --- | --- |
| *Individual Estimates from the Childhood Temperament-Only, Adult-Based Personality Only, and Combined Models Without Covariates for Education, Career, and Financial Outcomes* | | | | | | | | | |
|  |  | Highest Degree | | Employed | | Annual Salary | | Ever Receive Welfare | |
| Model | | *b* | *CI* | *OR* | *CI* | *b* | *CI* | *OR* | *CI* |
| Temperament | | | | | | | | | |
|  | Activity | 0.00 | [-0.09, 0.09] | 0.97 | [0.87, 1.07] | -0.01 | [-0.03, 0.01] | 1.16 | [0.95, 1.43] |
|  | Compliance | **0.21** | **[0.11, 0.30]** | 1.12 | [1.00, 1.26] | 0.01 | [-0.01, 0.04] | 0.82 | [0.67, 1.03] |
|  | Fearful | -0.07 | [-0.17, 0.03] | 0.99 | [0.88, 1.11] | -0.02 | [-0.04, 0.00] | 1.15 | [0.92, 1.42] |
|  | Insecure | **-0.11** | **[-0.21, -0.03]** | **0.89** | **[0.79, 0.99]** | -0.02 | [-0.05, 0.00] | **1.41** | **[1.14, 1.75]** |
|  | Pos. Affect | -0.06 | [-0.13, 0.02] | 0.97 | [0.89, 1.07] | **-0.03** | **[-0.05, -0.01]** | 0.88 | [0.74, 1.04] |
|  | Predictability | **0.16** | **[0.08, 0.25]** | 1.05 | [0.94, 1.16] | 0.02 | [-0.00, 0.04] | 0.90 | [0.74, 1.10] |
|  | Sociability | **0.21** | **[0.12, 0.30]** | 1.10 | [0.98, 1.23] | **0.03** | **[0.01, 0.06]** | **0.69** | **[0.56, 0.85]** |
| Personality | | | | | | | | | |
|  | E | **0.17** | **[0.12, 0.21]** | **1.15** | **[1.09, 1.22]** | **0.08** | **[0.06, 0.10]** | **0.77** | **[0.71, 0.84]** |
|  | A | **0.12** | **[0.08, 0.17]** | 1.01 | [0.95, 1.07] | **-0.03** | **[-0.05, -0.01]** | **1.21** | **[1.11, 1.33]** |
|  | C | **0.08** | **[0.04, 0.13]** | **1.09** | **[1.03, 1.16]** | **0.07** | **[0.05, 0.09]** | 0.98 | [0.90, 1.06] |
|  | N | **-0.16** | **[-0.21, -0.11]** | **0.89** | **[0.84, 0.94]** | **-0.03** | **[-0.05, -0.01]** | **1.34** | **[1.23, 1.46]** |
|  | O | -0.04 | [-0.09, 0.01] | 1.04 | [0.98, 1.10] | -0.01 | [-0.03, 0.00] | **0.83** | **[0.76, 0.91]** |
| Combined | | | | | | | | | |
|  | Activity | 0.01 | [-0.07, 0.10] | 0.97 | [0.88, 1.08] | -0.01 | [-0.03, 0.02] | 1.11 | [0.90, 1.38] |
|  | Compliance | **0.19** | **[0.09, 0.29]** | 1.11 | [0.99, 1.25] | 0.01 | [-0.01, 0.04] | 0.82 | [0.66, 1.03] |
|  | Fearful | -0.07 | [-0.17, 0.03] | 0.99 | [0.88, 1.11] | -0.02 | [-0.04, 0.01] | 1.11 | [0.89, 1.37] |
|  | Insecure | **-0.10** | **[-0.19, -0.01]** | 0.89 | [0.79, 1.00] | -0.02 | [-0.04, 0.00] | **1.37** | **[1.11, 1.70]** |
|  | Pos. Affect | -0.06 | [-0.14, 0.02] | 0.96 | [0.88, 1.06] | **-0.03** | **[-0.05, -0.01]** | 0.90 | [0.76, 1.07] |
|  | Predictability | **0.14** | **[0.04, 0.23]** | 1.03 | [0.93, 1.15] | 0.01 | [-0.01, 0.04] | 0.92 | [0.76, 1.13] |
|  | Sociability | **0.21** | **[0.11, 0.30]** | 1.11 | [0.99, 1.25] | **0.03** | **[0.01, 0.05]** | **0.71** | **[0.58, 0.88]** |
|  | E | 0.10 | [0.00, 0.19] | 1.11 | [0.99, 1.24] | **0.04** | **[0.02, 0.06]** | **0.79** | **[0.63, 0.98]** |
|  | A | **0.21** | **[0.12, 0.31]** | 1.02 | [0.91, 1.15] | -0.01 | [-0.04, 0.01] | 1.01 | [0.81, 1.26] |
|  | C | **0.12** | **[0.02, 0.21]** | 1.09 | [0.98, 1.21] | 0.02 | [-0.00, 0.04] | 0.96 | [0.78, 1.18] |
|  | N | **-0.17** | **[-0.27, -0.08]** | 0.97 | [0.87, 1.08] | -0.02 | [-0.04, 0.00] | **1.45** | **[1.17, 1.78]** |
|  | O | -0.05 | [-0.14, 0.04] | 1.08 | [0.97, 1.21] | 0.02 | [-0.01, 0.04] | **0.77** | **[0.61, 0.96]** |
| *Note*. *OR* = odds ratio. *b* = regression coefficient. *CI* = 95% credible interval. E = extraversion. A = agreeableness. C = conscientiousness. N = neuroticism. O = openness. Pos. Affect = positive affect. Bold values indicate that the credible intervals either do not contain 1.00 (for *OR*s) or do not contain 0.00 (for regression coefficients). Results are from models without covariates. All traits are standardized. | | | | | | | | | |

| **Table S7** | | | | | |
| --- | --- | --- | --- | --- | --- |
| *Individual Estimates from the Childhood Temperament-Only, Adult-Based Personality Only, and Combined Models Without Covariates for Civic Engagement Outcomes* | | | | | |
|  |  | Ever Volunteer | | Religious | |
| Model | | *OR* | *CI* | *OR* | *CI* |
| Temperament | | | | | |
|  | Activity | 0.92 | [0.82, 1.03] | 0.93 | [0.68, 1.25] |
|  | Compliance | **1.16** | **[1.02, 1.32]** | 0.90 | [0.65, 1.23] |
|  | Fearful | 0.94 | [0.83, 1.07] | 1.31 | [0.91, 1.92] |
|  | Insecure | 0.91 | [0.80, 1.03] | 1.18 | [0.86, 1.62] |
|  | Pos. Affect | 1.02 | [0.92, 1.13] | 1.00 | [0.77, 1.27] |
|  | Predictability | 1.08 | [0.96, 1.21] | 1.10 | [0.82, 1.47] |
|  | Sociability | **1.29** | **[1.14, 1.46]** | 1.10 | [0.80, 1.50] |
| Personality | | | | | |
|  | E | **1.30** | **[1.23, 1.38]** | 1.10 | [0.94, 1.29] |
|  | A | **1.13** | **[1.06, 1.19]** | **1.23** | **[1.06, 1.44]** |
|  | C | 1.03 | [0.97, 1.09] | 1.11 | [0.94, 1.29] |
|  | N | 0.95 | [0.89, 1.00] | 1.08 | [0.92, 1.27] |
|  | O | 1.06 | [1.00, 1.13] | 0.97 | [0.82, 1.13] |
| Combined | | | | | |
|  | Activity | 0.93 | [0.83, 1.05] | 0.93 | [0.69, 1.26] |
|  | Compliance | **1.15** | **[1.01, 1.31]** | 0.88 | [0.64, 1.20] |
|  | Fearful | 0.95 | [0.84, 1.08] | 1.32 | [0.92, 1.95] |
|  | Insecure | 0.92 | [0.81, 1.04] | 1.23 | [0.90, 1.71] |
|  | Pos. Affect | 1.02 | [0.92, 1.13] | 1.03 | [0.80, 1.31] |
|  | Predictability | 1.06 | [0.94, 1.19] | 1.06 | [0.79, 1.41] |
|  | Sociability | **1.27** | **[1.12, 1.44]** | 1.11 | [0.82, 1.53] |
|  | E | **1.20** | **[1.06, 1.36]** | 1.18 | [0.88, 1.59] |
|  | A | **1.15** | **[1.01, 1.31]** | **1.46** | **[1.10, 1.93]** |
|  | C | 1.14 | [1.00, 1.29] | 1.08 | [0.79, 1.44] |
|  | N | 0.92 | [0.82, 1.05] | 0.88 | [0.67, 1.19] |
|  | O | 1.04 | [0.92, 1.18] | **0.64** | **[0.45, 0.88]** |
| *Note*. *OR* = odds ratio. *CI* = 95% credible interval. E = extraversion. A = agreeableness. C = conscientiousness. N = neuroticism. O = openness. Pos. Affect = positive affect. Bold values indicate that the credible intervals do not contain 1.00. Results are from models without covariates. All traits are standardized. | | | | | |

| **Table S8a** | | | | | |
| --- | --- | --- | --- | --- | --- |
| *Individual Estimates from the Childhood Temperament-Only Models with Covariates for Health Outcomes* | | | | | |
|  |  | Health Status | | BMI | |
| Predictor | | *b* | *CI* | *b* | *CI* |
| Temperament | | | | | |
|  | Activity | -0.07 | [-0.16, 0.03] | 0.03 | [-0.01, 0.07] |
|  | Compliance | **0.13** | **[0.03, 0.23]** | **-0.06** | **[-0.10, -0.02]** |
|  | Fearful | -0.04 | [-0.15, 0.06] | -0.01 | [-0.05, 0.04] |
|  | Insecure | -0.04 | [-0.14, 0.07] | -0.01 | [-0.06, 0.03] |
|  | Pos. Affect | 0.01 | [-0.07, 0.09] | 0.01 | [-0.02, 0.05] |
|  | Predictability | -0.05 | [-0.15, 0.04] | -0.02 | [-0.06, 0.02] |
|  | Sociability | -0.01 | [-0.11, 0.09] | 0.00 | [-0.04, 0.04] |
| Covariates | | | | | |
|  | Age | -0.03 | [-0.07, 0.00] | **0.06** | **[0.04, 0.07]** |
|  | Gender | **-0.22** | **[-0.41, -0.02]** | -0.05 | [-0.13, 0.04] |
|  | Race | -0.01 | [-0.13, 0.10] | 0.04 | [-0.01, 0.09] |
|  | Mom age | 0.01 | [-0.02, 0.05] | 0.00 | [-0.01, 0.02] |
|  | Gestation time | 0.00 | [-0.05, 0.06] | 0.00 | [-0.02, 0.03] |
|  | Birth length | 0.02 | [-0.10, 0.13] | -0.02 | [-0.06, 0.03] |
|  | Birth weight | 0.01 | [-0.11, 0.13] | **0.07** | **[0.02, 0.12]** |
|  | Breastfed | 0.18 | [-0.01, 0.38] | **-0.08** | **[-0.17, -0.01]** |
|  | Substance use | -0.05 | [-0.23, 0.14] | -0.01 | [-0.09, 0.07] |
|  | Mom edu | **0.06** | **[0.02, 0.10]** | -0.01 | [-0.03, 0.01] |
| *Note*. *b* = regression coefficient. *CI* = 95% credible interval. E = extraversion. A = agreeableness. C = conscientiousness. N = neuroticism. O = openness. Mom age = mom age at birth of child. Gestation time = weeks that mother was pregnant with child. Substance use = if mother reported any substance use during pregnancy. Mom edu = highest education level achieved by mother. Bold values indicate that the credible intervals do not contain 0.00. Results are from models with covariates. All traits are standardized. | | | | | |

| **Table S8b** | | | | | |
| --- | --- | --- | --- | --- | --- |
| *Individual Estimates from the Adult-Based Personality-Only Models with Covariates for Health Outcomes* | | | | | |
|  |  | Health Status | | BMI | |
| Predictor | | *b* | *CI* | *b* | *CI* |
| Personality | | | | | |
|  | E | **0.13** | **[0.08, 0.18]** | 0.02 | [-0.00, 0.04] |
|  | A | 0.00 | [-0.05, 0.05] | -0.01 | [-0.03, 0.02] |
|  | C | **0.24** | **[0.19, 0.30]** | -0.01 | [-0.03, 0.02] |
|  | N | **-0.26** | **[-0.32, -0.20]** | 0.02 | [-0.01, 0.04] |
|  | O | 0.00 | [-0.06, 0.05] | 0.00 | [-0.02, 0.03] |
| Covariates | | | | | |
|  | Age | **-0.05** | **[-0.07, -0.03]** | **0.05** | **[0.04, 0.06]** |
|  | Gender | **-0.13** | **[-0.24, -0.03]** | -0.04 | [-0.09, 0.01] |
|  | Race | **-0.08** | **[-0.14, -0.02]** | **0.07** | **[0.04, 0.10]** |
|  | Mom age | 0.00 | [-0.02, 0.01] | 0.00 | [-0.00, 0.01] |
|  | Gestation time | 0.00 | [-0.02, 0.03] | 0.00 | [-0.01, 0.01] |
|  | Birth length | 0.01 | [-0.05, 0.06] | -0.02 | [-0.04, 0.01] |
|  | Birth weight | 0.02 | [-0.04, 0.08] | **0.05** | **[0.02, 0.08]** |
|  | Breastfed | **0.24** | **[0.14, 0.34]** | **-0.15** | **[-0.20, -0.11]** |
|  | Substance use | 0.00 | [-0.11, 0.10] | -0.04 | [-0.09, 0.00] |
|  | Mom edu | **0.06** | **[0.04, 0.08]** | -0.01 | [-0.02, 0.00] |
| *Note*. *b* = regression coefficient. *CI* = 95% credible interval. E = extraversion. A = agreeableness. C = conscientiousness. N = neuroticism. O = openness. Mom age = mom age at birth of child. Gestation time = weeks that mother was pregnant with child. Substance use = if mother reported any substance use during pregnancy. Mom edu = highest education level achieved by mother. Bold values indicate that the credible intervals do not contain 0.00. Results are from models with covariates. All traits are standardized. | | | | | |

| **Table S8c** | | | | | |
| --- | --- | --- | --- | --- | --- |
| *Individual Estimates from the Combined Models with Covariates for Health Outcomes* | | | | | |
|  |  | Health Status | | BMI | |
| Predictor | | *b* | *CI* | *b* | *CI* |
| Temperament | | | | | |
|  | Activity | -0.07 | [-0.17, 0.03] | 0.03 | [-0.01, 0.07] |
|  | Compliance | 0.11 | [0.00, 0.21] | **-0.06** | **[-0.10, -0.02]** |
|  | Fearful | -0.05 | [-0.16, 0.06] | 0.00 | [-0.05, 0.04] |
|  | Insecure | -0.05 | [-0.16, 0.05] | -0.01 | [-0.06, 0.03] |
|  | Pos. Affect | -0.01 | [-0.09, 0.07] | 0.02 | [-0.02, 0.05] |
|  | Predictability | -0.07 | [-0.17, 0.03] | -0.02 | [-0.07, 0.02] |
|  | Sociability | -0.01 | [-0.11, 0.10] | 0.00 | [-0.04, 0.05] |
| Personality | | | | | |
|  | E | **0.15** | **[0.05, 0.25]** | **0.06** | **[0.02, 0.10]** |
|  | A | -0.03 | [-0.13, 0.07] | 0.04 | [-0.01, 0.08] |
|  | C | **0.31** | **[0.21, 0.41]** | 0.00 | [-0.04, 0.05] |
|  | N | **-0.25** | **[-0.35, -0.15]** | **0.10** | **[0.05, 0.14]** |
|  | O | -0.06 | [-0.16, 0.04] | 0.01 | [-0.03, 0.05] |
| Covariates | | | | | |
|  | Age | **-0.06** | **[-0.10, -0.01]** | **0.05** | **[0.04, 0.07]** |
|  | Gender | -0.15 | [-0.35, 0.06] | **-0.11** | **[-0.20, -0.02]** |
|  | Race | 0.00 | [-0.12, 0.12] | 0.04 | [-0.00, 0.09] |
|  | Mom age | 0.01 | [-0.03, 0.04] | 0.00 | [-0.01, 0.02] |
|  | Gestation time | -0.01 | [-0.07, 0.05] | 0.00 | [-0.02, 0.03] |
|  | Birth length | 0.01 | [-0.11, 0.12] | -0.02 | [-0.07, 0.02] |
|  | Birth weight | 0.02 | [-0.10, 0.15] | **0.07** | **[0.02, 0.12]** |
|  | Breastfed | **0.20** | **[0.01, 0.39]** | -0.08 | [-0.17, 0.01] |
|  | Substance use | 0.04 | [-0.15, 0.23] | -0.02 | [-0.10, 0.06] |
|  | Mom edu | **0.06** | **[0.02, 0.10]** | -0.01 | [-0.03, 0.01] |
| *Note*. *b* = regression coefficient. *CI* = 95% credible interval. E = extraversion. A = agreeableness. C = conscientiousness. N = neuroticism. O = openness. Mom age = mom age at birth of child. Gestation time = weeks that mother was pregnant with child. Substance use = if mother reported any substance use during pregnancy. Mom edu = highest education level achieved by mother. Bold values indicate that the credible intervals do not contain 0.00. Results are from models with covariates. All traits are standardized. | | | | | |

| **Table S9a** | | | | | | | | | |
| --- | --- | --- | --- | --- | --- | --- | --- | --- | --- |
| *Individual Estimates from the Childhood Temperament-Only Models with Covariates for Internalizing Outcomes* | | | | | | | | | |
|  |  | Anxiety | | Depression | | Counselor | | Suicide | |
| Predictor | | *OR* | *CI* | *OR* | *CI* | *OR* | *CI* | *OR* | *CI* |
| Temperament | | | | | | | | | |
|  | Activity | 1.34 | [1.00, 1.79] | 1.16 | [0.84, 1.58] | 1.00 | [0.89, 1.11] | 0.95 | [0.80, 1.12] |
|  | Compliance | 0.92 | [0.67, 1.27] | 0.98 | [0.71, 1.36] | **0.76** | **[0.68, 0.86]** | 0.91 | [0.77, 1.09] |
|  | Fearful | 1.18 | [0.84, 1.66] | 1.04 | [0.71, 1.49] | 1.02 | [0.90, 1.16] | 1.05 | [0.88, 1.25] |
|  | Insecure | 0.84 | [0.60, 1.15] | 1.34 | [0.96, 1.88] | 1.03 | [0.91, 1.16] | 1.00 | [0.83, 1.20] |
|  | Pos. Affect | 0.97 | [0.74, 1.29] | 0.95 | [0.71, 1.28] | 1.05 | [0.96, 1.15] | 0.98 | [0.85, 1.13] |
|  | Predictability | 1.35 | [0.95, 2.00] | 0.93 | [0.66, 1.34] | 1.00 | [0.90, 1.12] | 1.02 | [0.86, 1.20] |
|  | Sociability | 1.01 | [0.73, 1.41] | **1.49** | **[1.05, 2.15]** | 1.03 | [0.91, 1.16] | 1.17 | [0.99, 1.40] |
| Covariates | | | | | | | | | |
|  | Age | 1.00 | [0.86, 1.16] | 1.05 | [0.90, 1.23] | 1.03 | [0.99, 1.07] | 1.04 | [0.97, 1.11] |
|  | Gender | 1.71 | [0.90, 3.25] | 1.48 | [0.77, 2.87] | **1.31** | **[1.07, 1.61]** | 1.35 | [0.98, 1.87] |
|  | Race | 0.72 | [0.49, 1.04] | **0.63** | **[0.40, 0.95]** | 0.96 | [0.84, 1.09] | 0.99 | [0.81, 1.21] |
|  | Mom age | 0.97 | [0.84, 1.12] | 1.06 | [0.91, 1.22] | 0.99 | [0.95, 1.03] | 1.00 | [0.94, 1.07] |
|  | Gestation time | 0.90 | [0.74, 1.10] | 0.84 | [0.68, 1.04] | 1.02 | [0.96, 1.09] | 1.03 | [0.93, 1.14] |
|  | Birth length | 1.07 | [0.68, 1.67] | 0.86 | [0.52, 1.42] | 0.96 | [0.85, 1.09] | 0.96 | [0.79, 1.19] |
|  | Birth weight | 1.18 | [0.75, 1.90] | 1.14 | [0.68, 1.90] | 0.91 | [0.79, 1.05] | 1.03 | [0.84, 1.28] |
|  | Breastfed | 1.62 | [0.84, 3.14] | **2.78** | **[1.40, 5.77]** | **1.26** | **[1.02, 1.57]** | 0.96 | [0.68, 1.35] |
|  | Substance use | 1.66 | [0.86, 3.25] | 1.93 | [0.97, 4.01] | **1.35** | **[1.09, 1.69]** | **1.42** | **[1.01, 2.01]** |
|  | Mom edu | 1.03 | [0.91, 1.16] | 0.97 | [0.85, 1.11] | 1.04 | [1.00, 1.09] | 0.96 | [0.90, 1.03] |
| *Note*. *OR* = odds ratio. *CI* = 95% credible interval. E = extraversion. A = agreeableness. C = conscientiousness. N = neuroticism. O = openness. Mom age = mom age at birth of child. Gestation time = weeks that mother was pregnant with child. Substance use = if mother reported any substance use during pregnancy. Mom edu = highest education level achieved by mother. Bold values indicate that the credible intervals do not contain 1.00. Results are from models with covariates. All traits are standardized. | | | | | | | | | |

| **Table S9b** | | | | | | | | | |
| --- | --- | --- | --- | --- | --- | --- | --- | --- | --- |
| *Individual Estimates from the Adult-Based Personality-Only Models with Covariates for Internalizing Outcomes* | | | | | | | | | |
|  |  | Anxiety | | Depression | | Counselor | | Suicide | |
| Predictor | | *OR* | *CI* | *OR* | *CI* | *OR* | *CI* | *OR* | *CI* |
| Personality | | | | | | | | | |
|  | E | 0.87 | [0.75, 1.02] | **0.79** | **[0.67, 0.92]** | 1.02 | [0.96, 1.08] | **0.87** | **[0.79, 0.95]** |
|  | A | 1.19 | [1.00, 1.42] | 1.10 | [0.92, 1.33] | 1.05 | [0.98, 1.11] | **1.20** | **[1.09, 1.33]** |
|  | C | **1.23** | **[1.04, 1.46]** | 1.17 | [0.99, 1.37] | **0.82** | **[0.77, 0.87]** | **0.85** | **[0.77, 0.93]** |
|  | N | **1.52** | **[1.28, 1.78]** | **1.53** | **[1.30, 1.80]** | **1.51** | **[1.42, 1.61]** | **2.04** | **[1.86, 2.25]** |
|  | O | 1.18 | [0.99, 1.41] | **1.22** | **[1.03, 1.45]** | **1.13** | **[1.07, 1.21]** | **1.35** | **[1.22, 1.49]** |
| Covariates | | | | | | | | | |
|  | Age | 0.93 | [0.87, 1.00] | 1.00 | [0.93, 1.07] | **1.03** | **[1.01, 1.05]** | 1.02 | [0.99, 1.06] |
|  | Gender | **1.99** | **[1.38, 2.91]** | **1.64** | **[1.16, 2.34]** | **1.17** | **[1.04, 1.32]** | 1.04 | [0.85, 1.26] |
|  | Race | **0.77** | **[0.62, 0.94]** | **0.77** | **[0.62, 0.94]** | 1.00 | [0.94, 1.08] | 1.03 | [0.92, 1.15] |
|  | Mom age | 0.99 | [0.93, 1.06] | 1.01 | [0.95, 1.08] | 1.01 | [0.99, 1.03] | 1.03 | [0.99, 1.06] |
|  | Gestation time | 0.97 | [0.89, 1.07] | 0.96 | [0.88, 1.06] | 1.03 | [1.00, 1.07] | 1.00 | [0.95, 1.06] |
|  | Birth length | 1.15 | [0.92, 1.46] | 1.00 | [0.80, 1.26] | 0.99 | [0.94, 1.06] | 0.98 | [0.88, 1.10] |
|  | Birth weight | 1.03 | [0.83, 1.28] | 1.07 | [0.87, 1.32] | **0.90** | **[0.85, 0.97]** | 1.01 | [0.90, 1.14] |
|  | Breastfed | 1.30 | [0.93, 1.83] | **1.42** | **[1.01, 2.00]** | **1.16** | **[1.04, 1.31]** | 1.07 | [0.88, 1.28] |
|  | Substance use | 0.84 | [0.59, 1.18] | 1.07 | [0.77, 1.50] | **1.26** | **[1.13, 1.40]** | **1.27** | **[1.06, 1.53]** |
|  | Mom edu | 0.96 | [0.89, 1.02] | 1.00 | [0.93, 1.06] | **1.04** | **[1.02, 1.06]** | 0.99 | [0.95, 1.03] |
| *Note*. *OR* = odds ratio. *CI* = 95% credible interval. E = extraversion. A = agreeableness. C = conscientiousness. N = neuroticism. O = openness. Mom age = mom age at birth of child. Gestation time = weeks that mother was pregnant with child. Substance use = if mother reported any substance use during pregnancy. Mom edu = highest education level achieved by mother. Bold values indicate that the credible intervals do not contain 1.00. Results are from models with covariates. All traits are standardized. | | | | | | | | | |

| **Table S9c** | | | | | | | | | |
| --- | --- | --- | --- | --- | --- | --- | --- | --- | --- |
| *Individual Estimates from the Combined Models with Covariates for Internalizing Outcomes* | | | | | | | | | |
|  |  | Anxiety | | Depression | | Counselor | | Suicide | |
| Predictor | | *OR* | *CI* | *OR* | *CI* | *OR* | *CI* | *OR* | *CI* |
| Temperament | | | | | | | | | |
|  | Activity | **1.38** | **[1.01, 1.88]** | 1.17 | [0.81, 1.69] | 0.98 | [0.87, 1.10] | 0.91 | [0.76, 1.07] |
|  | Compliance | 0.93 | [0.67, 1.29] | 0.89 | [0.62, 1.30] | **0.76** | **[0.68, 0.86]** | 0.93 | [0.77, 1.12] |
|  | Fearful | 1.18 | [0.83, 1.69] | 1.02 | [0.69, 1.53] | 1.01 | [0.89, 1.15] | 1.04 | [0.86, 1.26] |
|  | Insecure | 0.78 | [0.56, 1.11] | 1.27 | [0.87, 1.88] | 1.00 | [0.88, 1.13] | 0.93 | [0.78, 1.12] |
|  | Pos. Affect | 0.97 | [0.71, 1.35] | 1.00 | [0.70, 1.41] | 1.06 | [0.97, 1.17] | 1.01 | [0.87, 1.18] |
|  | Predictability | 1.36 | [0.91, 2.05] | 1.04 | [0.68, 1.60] | 1.02 | [0.91, 1.14] | 1.09 | [0.92, 1.30] |
|  | Sociability | 0.95 | [0.67, 1.36] | **1.66** | **[1.09, 2.52]** | 1.02 | [0.91, 1.15] | 1.20 | [0.99, 1.46] |
| Personality | | | | | | | | | |
|  | E | 1.14 | [0.80, 1.64] | **0.54** | **[0.36, 0.82]** | 1.00 | [0.89, 1.12] | **0.77** | **[0.65, 0.92]** |
|  | A | 1.40 | [0.94, 2.04] | **1.70** | **[1.11, 2.67]** | 1.01 | [0.89, 1.15] | 1.10 | [0.90, 1.34] |
|  | C | 1.26 | [0.92, 1.77] | **1.49** | **[1.03, 2.18]** | **0.86** | **[0.76, 0.96]** | 0.97 | [0.81, 1.16] |
|  | N | **1.68** | **[1.23, 2.29]** | **2.36** | **[1.65, 3.42]** | **1.68** | **[1.48, 1.90]** | **2.20** | **[1.83, 2.65]** |
|  | O | 1.12 | [0.80, 1.62] | **1.70** | **[1.14, 2.60]** | **1.24** | **[1.10, 1.41]** | **1.36** | **[1.13, 1.63]** |
| Covariates | | | | | | | | | |
|  | Age | 0.99 | [0.85, 1.16] | 1.08 | [0.91, 1.28] | 1.04 | [1.00, 1.09] | 1.06 | [0.98, 1.14] |
|  | Gender | 1.07 | [0.52, 2.18] | 0.66 | [0.29, 1.46] | 1.06 | [0.84, 1.35] | 0.89 | [0.62, 1.27] |
|  | Race | 0.75 | [0.51, 1.11] | 0.66 | [0.40, 1.02] | 0.94 | [0.83, 1.07] | 1.00 | [0.80, 1.24] |
|  | Mom age | 0.96 | [0.83, 1.11] | 1.04 | [0.89, 1.21] | 0.99 | [0.95, 1.04] | 1.01 | [0.94, 1.08] |
|  | Gestation time | 0.89 | [0.71, 1.11] | 0.80 | [0.63, 1.02] | 1.02 | [0.95, 1.09] | 1.03 | [0.92, 1.14] |
|  | Birth length | 1.11 | [0.66, 1.89] | 1.12 | [0.63, 1.99] | 0.97 | [0.84, 1.11] | 1.02 | [0.82, 1.27] |
|  | Birth weight | 1.14 | [0.69, 1.88] | 0.99 | [0.55, 1.79] | 0.89 | [0.77, 1.03] | 1.00 | [0.80, 1.27] |
|  | Breastfed | 1.63 | [0.82, 3.17] | **2.87** | **[1.30, 6.54]** | **1.32** | **[1.05, 1.65]** | 1.05 | [0.73, 1.49] |
|  | Substance use | 1.48 | [0.74, 3.11] | 1.76 | [0.79, 4.00] | 1.23 | [0.98, 1.54] | 1.24 | [0.88, 1.77] |
|  | Mom edu | 1.02 | [0.90, 1.16] | 0.96 | [0.82, 1.11] | 1.04 | [1.00, 1.09] | 0.96 | [0.89, 1.03] |
| *Note*. *OR* = odds ratio. *CI* = 95% credible interval. E = extraversion. A = agreeableness. C = conscientiousness. N = neuroticism. O = openness. Mom age = mom age at birth of child. Gestation time = weeks that mother was pregnant with child. Substance use = if mother reported any substance use during pregnancy. Mom edu = highest education level achieved by mother. Bold values indicate that the credible intervals do not contain 1.00. Results are from models with covariates. All traits are standardized. | | | | | | | | | |

| **Table S10a** | | | | | | | |
| --- | --- | --- | --- | --- | --- | --- | --- |
| *Individual Estimates from the Childhood Temperament-Only Models with Covariates for Externalizing Outcomes* | | | | | | | |
|  |  | ADHD | | Ever Been in Jail | | Number of Substances | |
| Predictor | | *OR* | *CI* | *OR* | *CI* | *IRR* | *CI* |
| Temperament | | | | | | | |
|  | Activity | 0.91 | [0.66, 1.23] | 0.90 | [0.71, 1.14] | 0.98 | [0.95, 1.01] |
|  | Compliance | **0.58** | **[0.43, 0.79]** | 1.00 | [0.79, 1.27] | 1.00 | [0.96, 1.03] |
|  | Fearful | 0.76 | [0.53, 1.07] | 1.06 | [0.84, 1.32] | 1.00 | [0.97, 1.04] |
|  | Insecure | 0.79 | [0.56, 1.11] | 1.11 | [0.89, 1.39] | **0.96** | **[0.93, 0.99]** |
|  | Pos. Affect | 1.01 | [0.79, 1.29] | **1.24** | **[1.01, 1.52]** | 0.99 | [0.96, 1.01] |
|  | Predictability | 1.14 | [0.84, 1.58] | **0.77** | **[0.63, 0.96]** | 1.02 | [0.99, 1.06] |
|  | Sociability | 0.87 | [0.64, 1.17] | 0.87 | [0.69, 1.09] | 1.02 | [0.99, 1.06] |
| Covariates | | | | | | | |
|  | Age | 0.90 | [0.81, 1.02] | 0.90 | [0.71, 1.14] | 0.98 | [0.95, 1.01] |
|  | Gender | 0.64 | [0.34, 1.18] | 1.00 | [0.79, 1.27] | 1.00 | [0.96, 1.03] |
|  | Race | 0.95 | [0.67, 1.33] | 1.06 | [0.84, 1.32] | 1.00 | [0.97, 1.04] |
|  | Mom age | 0.95 | [0.84, 1.06] | 1.11 | [0.89, 1.39] | **0.96** | **[0.93, 0.99]** |
|  | Gestation time | 1.12 | [0.94, 1.35] | **1.24** | **[1.01, 1.52]** | 0.99 | [0.96, 1.01] |
|  | Birth length | 1.06 | [0.71, 1.58] | **0.77** | **[0.63, 0.96]** | 1.02 | [0.99, 1.06] |
|  | Birth weight | 0.91 | [0.60, 1.35] | 0.87 | [0.69, 1.09] | 1.02 | [0.99, 1.06] |
|  | Breastfed | 0.92 | [0.50, 1.72] | 0.90 | [0.71, 1.14] | 0.98 | [0.95, 1.01] |
|  | Substance use | **2.36** | **[1.22, 4.72]** | 1.00 | [0.79, 1.27] | 1.00 | [0.96, 1.03] |
|  | Mom edu | 1.07 | [0.95, 1.21] | 1.06 | [0.84, 1.32] | 1.00 | [0.97, 1.04] |
| *Note*. *OR* = odds ratio. *IRR* = incidence rate ratio. *CI* = 95% credible interval. Pos. Affect = positive affect. Mom age = mom age at birth of child. Gestation time = weeks that mother was pregnant with child. Substance use = if mother reported any substance use during pregnancy. Mom edu = highest education level achieved by mother. Bold values indicate that the credible intervals do not contain 1.00. Results are from models with covariates. All traits are standardized. | | | | | | | |

| **Table S10b** | | | | | | | |
| --- | --- | --- | --- | --- | --- | --- | --- |
| *Individual Estimates from the Adult-Based Personality-Only Models with Covariates for Externalizing Outcomes* | | | | | | | |
|  |  | ADHD | | Ever Been in Jail | | Number of Substances | |
| Predictor | | *OR* | *CI* | *OR* | *CI* | *IRR* | *CI* |
| Personality | | | | | | | |
|  | E | 1.17 | [1.00, 1.37] | **0.86** | **[0.78, 0.95]** | **1.06** | **[1.04, 1.07]** |
|  | A | 1.04 | [0.88, 1.24] | **0.89** | **[0.80, 0.97]** | 0.98 | [0.97, 1.00] |
|  | C | **0.85** | **[0.73, 0.98]** | 1.03 | [0.93, 1.14] | **0.93** | **[0.91, 0.94]** |
|  | N | **1.30** | **[1.11, 1.53]** | **1.36** | **[1.23, 1.51]** | **1.09** | **[1.08, 1.11]** |
|  | O | 1.00 | [0.85, 1.17] | **1.20** | **[1.08, 1.32]** | **1.07** | **[1.06, 1.09]** |
| Covariates | | | | | | | |
|  | Age | 0.96 | [0.91, 1.02] | **1.05** | **[1.02, 1.09]** | **1.03** | **[1.03, 1.04]** |
|  | Gender | **0.29** | **[0.20, 0.41]** | **0.16** | **[0.13, 0.21]** | **0.84** | **[0.81, 0.87]** |
|  | Race | 0.89 | [0.73, 1.08] | **1.34** | **[1.19, 1.53]** | 1.02 | [1.00, 1.04] |
|  | Mom age | 0.99 | [0.94, 1.04] | **0.91** | **[0.88, 0.94]** | 0.99 | [0.99, 1.00] |
|  | Gestation time | 1.03 | [0.95, 1.12] | 1.02 | [0.97, 1.08] | 1.00 | [0.99, 1.01] |
|  | Birth length | 1.05 | [0.87, 1.28] | 0.94 | [0.85, 1.04] | 1.02 | [1.00, 1.03] |
|  | Birth weight | 0.88 | [0.73, 1.06] | 0.96 | [0.85, 1.08] | 1.01 | [0.99, 1.03] |
|  | Breastfed | 1.14 | [0.83, 1.58] | **0.75** | **[0.61, 0.92]** | **1.07** | **[1.04, 1.11]** |
|  | Substance use | 1.10 | [0.80, 1.54] | **1.50** | **[1.23, 1.83]** | **1.14** | **[1.11, 1.18]** |
|  | Mom edu | 1.00 | [0.93, 1.07] | **0.89** | **[0.85, 0.92]** | **0.99** | **[0.98, 0.99]** |
| *Note*. *OR* = odds ratio. *IRR* = incidence rate ratio. *CI* = 95% credible interval. Pos. Affect = positive affect. Mom age = mom age at birth of child. Gestation time = weeks that mother was pregnant with child. Substance use = if mother reported any substance use during pregnancy. Mom edu = highest education level achieved by mother. Bold values indicate that the credible intervals do not contain 1.00. Results are from models with covariates. All traits are standardized. | | | | | | | |

| **Table S10c** | | | | | | | |
| --- | --- | --- | --- | --- | --- | --- | --- |
| *Individual Estimates from the Combined Models with Covariates for Externalizing Outcomes* | | | | | | | |
|  |  | ADHD | | Ever Been in Jail | | Number of Substances | |
| Predictor | | *OR* | *CI* | *OR* | *CI* | *IRR* | *CI* |
| Temperament | | | | | | | |
|  | Activity | 0.86 | [0.62, 1.18] | 0.90 | [0.72, 1.14] | 0.98 | [0.94, 1.01] |
|  | Compliance | **0.58** | **[0.42, 0.79]** | 1.01 | [0.80, 1.27] | 1.00 | [0.97, 1.03] |
|  | Fearful | 0.78 | [0.55, 1.12] | 1.06 | [0.85, 1.32] | 1.01 | [0.98, 1.05] |
|  | Insecure | 0.78 | [0.54, 1.11] | 1.06 | [0.85, 1.35] | **0.95** | **[0.92, 0.99]** |
|  | Pos. Affect | 1.01 | [0.79, 1.32] | 1.22 | [1.00, 1.51] | 0.99 | [0.96, 1.02] |
|  | Predictability | 1.12 | [0.83, 1.54] | **0.80** | **[0.65, 0.99]** | 1.02 | [0.99, 1.06] |
|  | Sociability | 0.82 | [0.61, 1.11] | 0.88 | [0.69, 1.11] | 1.02 | [0.98, 1.05] |
| Personality | | | | | | | |
|  | E | 1.29 | [0.94, 1.76] | **0.75** | **[0.60, 0.94]** | **1.07** | **[1.03, 1.10]** |
|  | A | 0.89 | [0.65, 1.24] | 0.81 | [0.64, 1.02] | 0.97 | [0.94, 1.01] |
|  | C | 0.90 | [0.67, 1.21] | 0.81 | [0.64, 1.03] | **0.92** | **[0.89, 0.95]** |
|  | N | 1.27 | [0.91, 1.75] | 1.24 | [0.97, 1.58] | **1.12** | **[1.08, 1.15]** |
|  | O | 0.86 | [0.63, 1.18] | **1.46** | **[1.13, 1.88]** | **1.09** | **[1.05, 1.13]** |
| Covariates | | | | | | | |
|  | Age | 0.90 | [0.80, 1.01] | 1.08 | [1.00, 1.18] | **1.06** | **[1.04, 1.07]** |
|  | Gender | 0.61 | [0.32, 1.19] | **0.14** | **[0.08, 0.25]** | **0.84** | **[0.78, 0.90]** |
|  | Race | 0.93 | [0.65, 1.34] | **1.59** | **[1.23, 2.05]** | 1.01 | [0.98, 1.05] |
|  | Mom age | 0.94 | [0.84, 1.06] | **0.90** | **[0.83, 0.97]** | 1.00 | [0.99, 1.02] |
|  | Gestation time | 1.11 | [0.93, 1.35] | 1.08 | [0.94, 1.23] | 1.00 | [0.98, 1.02] |
|  | Birth length | 1.06 | [0.70, 1.59] | **0.77** | **[0.61, 0.97]** | 1.02 | [0.98, 1.06] |
|  | Birth weight | 0.91 | [0.61, 1.40] | 0.95 | [0.72, 1.23] | 1.01 | [0.97, 1.05] |
|  | Breastfed | 0.90 | [0.48, 1.68] | 0.95 | [0.60, 1.48] | 1.06 | [0.99, 1.13] |
|  | Substance use | **2.20** | **[1.14, 4.42]** | 1.39 | [0.89, 2.18] | 1.05 | [0.99, 1.12] |
|  | Mom edu | 1.07 | [0.95, 1.22] | **0.86** | **[0.79, 0.95]** | **0.98** | **[0.96, 0.99]** |
| *Note*. *OR* = odds ratio. *IRR* = incidence rate ratio. *CI* = 95% credible interval. Pos. Affect = positive affect. Mom age = mom age at birth of child. Gestation time = weeks that mother was pregnant with child. Substance use = if mother reported any substance use during pregnancy. Mom edu = highest education level achieved by mother. Bold values indicate that the credible intervals do not contain 1.00. Results are from models with covariates. All traits are standardized. | | | | | | | |

| **Table S11a** | | | | | | | | | | | |
| --- | --- | --- | --- | --- | --- | --- | --- | --- | --- | --- | --- |
| *Individual Estimates from the Childhood Temperament-Only Models with Covariates for Cognitive Outcomes* | | | | | | | | | | | |
|  |  | Digit Span | | Word Recall | | PIAT Math | | PIAT Read Comp | | PIAT Read Rec | |
| Predictor | | *b* | *CI* | *b* | *CI* | *b* | *CI* | *b* | *CI* | *b* | *CI* |
| Temperament | | | | | | | | | | | |
|  | Activity | **-0.06** | **[-0.11, -0.01]** | **-0.19** | **[-0.36, -0.03]** | -0.03 | [-0.07, 0.01] | -0.02 | [-0.06, 0.02] | -0.04 | [-0.08, 0.00] |
|  | Compliance | **0.08** | **[0.03, 0.13]** | -0.03 | [-0.19, 0.13] | **0.11** | **[0.07, 0.16]** | **0.12** | **[0.08, 0.17]** | **0.11** | **[0.07, 0.16]** |
|  | Fearful | 0.03 | [-0.02, 0.09] | -0.06 | [-0.24, 0.12] | -0.02 | [-0.06, 0.03] | -0.01 | [-0.06, 0.04] | -0.02 | [-0.06, 0.03] |
|  | Insecure | -0.03 | [-0.08, 0.03] | -0.15 | [-0.33, 0.03] | -0.01 | [-0.06, 0.03] | -0.01 | [-0.05, 0.03] | -0.01 | [-0.06, 0.03] |
|  | Pos. Affect | 0.00 | [-0.04, 0.04] | 0.08 | [-0.06, 0.23] | 0.02 | [-0.02, 0.05] | -0.01 | [-0.04, 0.02] | -0.01 | [-0.05, 0.02] |
|  | Predictability | -0.01 | [-0.07, 0.04] | 0.05 | [-0.12, 0.21] | 0.03 | [-0.02, 0.07] | 0.03 | [-0.01, 0.07] | 0.04 | [0.00, 0.09] |
|  | Sociability | **0.15** | **[0.09, 0.20]** | 0.10 | [-0.07, 0.26] | **0.16** | **[0.12, 0.21]** | **0.18** | **[0.13, 0.22]** | **0.16** | **[0.11, 0.20]** |
| Covariates | | | | | | | | | | | |
|  | Age | **0.15** | **[0.10, 0.21]** | 0.01 | [-0.28, 0.29] | **0.28** | **[0.25, 0.30]** | **0.27** | **[0.24, 0.29]** | **0.25** | **[0.23, 0.28]** |
|  | Gender | 0.07 | [-0.03, 0.16] | -0.15 | [-0.46, 0.15] | -0.07 | [-0.15, 0.01] | **0.11** | **[0.03, 0.18]** | **0.12** | **[0.04, 0.20]** |
|  | Race | **-0.09** | **[-0.15, -0.02]** | -0.14 | [-0.33, 0.05] | **-0.19** | **[-0.23, -0.14]** | **-0.15** | **[-0.20, -0.11]** | **-0.11** | **[-0.16, -0.06]** |
|  | Mom age | 0.01 | [-0.01, 0.02] | -0.02 | [-0.08, 0.04] | **0.04** | **[0.03, 0.05]** | **0.02** | **[0.01, 0.03]** | **0.03** | **[0.02, 0.04]** |
|  | Gestation time | 0.00 | [-0.03, 0.03] | 0.01 | [-0.10, 0.12] | 0.01 | [-0.02, 0.03] | 0.00 | [-0.03, 0.02] | -0.01 | [-0.03, 0.02] |
|  | Birth length | 0.01 | [-0.05, 0.08] | 0.20 | [-0.01, 0.40] | 0.02 | [-0.03, 0.07] | 0.02 | [-0.03, 0.07] | 0.01 | [-0.04, 0.06] |
|  | Birth weight | 0.01 | [-0.05, 0.07] | -0.15 | [-0.37, 0.07] | 0.05 | [0.00, 0.11] | 0.05 | [-0.00, 0.10] | **0.07** | **[0.02, 0.13]** |
|  | Breastfed | 0.09 | [-0.01, 0.19] | 0.19 | [-0.13, 0.51] | **0.21** | **[0.12, 0.29]** | **0.24** | **[0.16, 0.32]** | **0.21** | **[0.12, 0.29]** |
|  | Substance use | 0.00 | [-0.10, 0.10] | 0.11 | [-0.19, 0.41] | **0.11** | **[0.04, 0.20]** | 0.06 | [-0.02, 0.13] | 0.02 | [-0.06, 0.10] |
|  | Mom edu | **0.05** | **[0.03, 0.07]** | 0.02 | [-0.05, 0.09] | **0.07** | **[0.06, 0.09]** | **0.07** | **[0.05, 0.08]** | **0.06** | **[0.05, 0.08]** |
| *Note*. *b* = regression coefficient. *CI* = 95% credible interval. Pos. Affect = positive affect. Mom age = mom age at birth of child. Gestation time = weeks that mother was pregnant with child. Substance use = if mother reported any substance use during pregnancy. Mom edu = highest education level achieved by mother. Bold values indicate that the credible intervals do not contain 0.00. Results are from models with covariates. All traits are standardized. | | | | | | | | | | | |

| **Table S11b** | | | | | | | | | | | |
| --- | --- | --- | --- | --- | --- | --- | --- | --- | --- | --- | --- |
| *Individual Estimates from the Adult-Based Personality-Only Models with Covariates for Cognitive Outcomes* | | | | | | | | | | | |
|  |  | Digit Span | | Word Recall | | PIAT Math | | PIAT Read Comp | | PIAT Read Rec | |
| Predictor | | *b* | *CI* | *b* | *CI* | *b* | *CI* | *b* | *CI* | *b* | *CI* |
| Personality | | | | | | | | | | | |
|  | E | **0.07** | **[0.04, 0.09]** | 0.06 | [-0.00, 0.12] | **0.06** | **[0.04, 0.08]** | **0.06** | **[0.03, 0.08]** | **0.05** | **[0.02, 0.07]** |
|  | A | -0.01 | [-0.04, 0.02] | 0.05 | [-0.02, 0.11] | 0.01 | [-0.01, 0.04] | **0.05** | **[0.03, 0.08]** | **0.05** | **[0.03, 0.08]** |
|  | C | -0.02 | [-0.04, 0.01] | 0.01 | [-0.06, 0.09] | **-0.06** | **[-0.09, -0.04]** | **-0.06** | **[-0.09, -0.04]** | **-0.04** | **[-0.07, -0.01]** |
|  | N | -0.01 | [-0.04, 0.01] | -0.03 | [-0.10, 0.04] | **-0.04** | **[-0.06, -0.02]** | -0.03 | [-0.05, -0.00] | -0.02 | [-0.05, 0.00] |
|  | O | **0.07** | **[0.04, 0.09]** | 0.03 | [-0.04, 0.09] | **0.03** | **[0.01, 0.05]** | **0.04** | **[0.02, 0.07]** | **0.06** | **[0.03, 0.08]** |
| Covariates | | | | | | | | | | | |
|  | Age | **0.15** | **[0.13, 0.17]** | -0.02 | [-0.06, 0.02] | **0.29** | **[0.27, 0.30]** | **0.26** | **[0.25, 0.28]** | **0.26** | **[0.25, 0.27]** |
|  | Gender | **0.12** | **[0.06, 0.17]** | **0.15** | **[0.02, 0.27]** | -0.03 | [-0.07, 0.02] | **0.11** | **[0.06, 0.15]** | **0.14** | **[0.09, 0.18]** |
|  | Race | **-0.10** | **[-0.13, -0.07]** | -0.04 | [-0.11, 0.04] | **-0.17** | **[-0.20, -0.14]** | **-0.13** | **[-0.16, -0.10]** | **-0.10** | **[-0.13, -0.07]** |
|  | Mom age | 0.01 | [0.00, 0.01] | -0.01 | [-0.03, 0.01] | **0.02** | **[0.02, 0.03]** | 0.00 | [-0.00, 0.01] | **0.01** | **[0.01, 0.02]** |
|  | Gestation time | 0.00 | [-0.01, 0.02] | 0.01 | [-0.02, 0.04] | 0.00 | [-0.01, 0.01] | 0.00 | [-0.01, 0.01] | 0.01 | [-0.00, 0.02] |
|  | Birth length | 0.01 | [-0.02, 0.04] | 0.00 | [-0.06, 0.07] | 0.01 | [-0.02, 0.03] | 0.01 | [-0.02, 0.03] | 0.00 | [-0.02, 0.02] |
|  | Birth weight | 0.03 | [-0.01, 0.06] | 0.03 | [-0.05, 0.10] | **0.06** | **[0.03, 0.08]** | **0.05** | **[0.02, 0.08]** | **0.04** | **[0.02, 0.07]** |
|  | Breastfed | **0.14** | **[0.09, 0.20]** | **0.18** | **[0.05, 0.30]** | **0.23** | **[0.19, 0.28]** | **0.24** | **[0.20, 0.29]** | **0.20** | **[0.15, 0.25]** |
|  | Substance use | 0.05 | [0.00, 0.10] | 0.04 | [-0.08, 0.16] | **0.12** | **[0.08, 0.16]** | **0.07** | **[0.02, 0.11]** | 0.04 | [-0.00, 0.09] |
|  | Mom edu | **0.06** | **[0.05, 0.08]** | **0.04** | **[0.02, 0.07]** | **0.07** | **[0.06, 0.08]** | **0.08** | **[0.07, 0.09]** | **0.07** | **[0.06, 0.08]** |
| *Note*. *b* = regression coefficient. *CI* = 95% credible interval. E = extraversion. A = agreeableness. C = conscientiousness. N = neuroticism. O = openness. Mom age = mom age at birth of child. Gestation time = weeks that mother was pregnant with child. Substance use = if mother reported any substance use during pregnancy. Mom edu = highest education level achieved by mother. Bold values indicate that the credible intervals do not contain 0.00. Results are from models with covariates. All traits are standardized. | | | | | | | | | | | |

| **Table S11c** | | | | | | | | | | | |
| --- | --- | --- | --- | --- | --- | --- | --- | --- | --- | --- | --- |
| *Individual Estimates from the Combined Models with Covariates for Cognitive Outcomes* | | | | | | | | | | | |
|  |  | Digit Span | | Word Recall | | PIAT Math | | PIAT Read Comp | | PIAT Read Rec | |
| Predictor | | *b* | *CI* | *b* | *CI* | *b* | *CI* | *b* | *CI* | *b* | *CI* |
| Temperament | | | | | | | | | | | |
|  | Activity | -0.05 | [-0.10, -0.00] | **-0.19** | **[-0.36, -0.02]** | -0.02 | [-0.06, 0.02] | -0.01 | [-0.05, 0.03] | -0.03 | [-0.07, 0.01] |
|  | Compliance | **0.08** | **[0.02, 0.13]** | -0.02 | [-0.19, 0.15] | **0.11** | **[0.07, 0.15]** | **0.12** | **[0.08, 0.16]** | **0.11** | **[0.06, 0.16]** |
|  | Fearful | 0.04 | [-0.02, 0.09] | -0.07 | [-0.26, 0.12] | -0.01 | [-0.06, 0.04] | -0.01 | [-0.05, 0.04] | -0.01 | [-0.06, 0.03] |
|  | Insecure | -0.03 | [-0.08, 0.02] | -0.16 | [-0.35, 0.03] | -0.01 | [-0.05, 0.04] | 0.00 | [-0.04, 0.04] | -0.01 | [-0.05, 0.04] |
|  | Pos. Affect | 0.00 | [-0.04, 0.04] | 0.08 | [-0.07, 0.23] | 0.02 | [-0.02, 0.05] | -0.01 | [-0.04, 0.02] | -0.01 | [-0.05, 0.02] |
|  | Predictability | -0.02 | [-0.07, 0.03] | 0.05 | [-0.11, 0.22] | 0.02 | [-0.02, 0.06] | 0.02 | [-0.02, 0.06] | 0.04 | [-0.01, 0.08] |
|  | Sociability | **0.14** | **[0.09, 0.19]** | 0.10 | [-0.06, 0.26] | **0.16** | **[0.11, 0.20]** | **0.17** | **[0.13, 0.21]** | **0.15** | **[0.11, 0.20]** |
| Personality | | | | | | | | | | | |
|  | E | **0.08** | **[0.03, 0.13]** | -0.06 | [-0.22, 0.11] | **0.07** | **[0.02, 0.11]** | **0.05** | **[0.01, 0.09]** | 0.03 | [-0.01, 0.07] |
|  | A | **-0.06** | **[-0.11, -0.01]** | 0.02 | [-0.16, 0.20] | -0.02 | [-0.06, 0.03] | 0.04 | [-0.00, 0.09] | 0.05 | [0.00, 0.09] |
|  | C | 0.00 | [-0.05, 0.05] | -0.02 | [-0.20, 0.16] | **-0.06** | **[-0.11, -0.02]** | **-0.05** | **[-0.09, -0.01]** | -0.03 | [-0.07, 0.02] |
|  | N | -0.03 | [-0.09, 0.02] | -0.04 | [-0.23, 0.14] | **-0.09** | **[-0.13, -0.05]** | -0.04 | [-0.08, 0.01] | -0.03 | [-0.07, 0.02] |
|  | O | **0.10** | **[0.05, 0.15]** | 0.00 | [-0.19, 0.19] | 0.04 | [-0.00, 0.09] | 0.03 | [-0.01, 0.08] | 0.05 | [0.00, 0.09] |
| Covariates | | | | | | | | | | | |
|  | Age | **0.15** | **[0.10, 0.21]** | -0.02 | [-0.31, 0.28] | **0.28** | **[0.25, 0.30]** | **0.27** | **[0.24, 0.29]** | **0.25** | **[0.23, 0.28]** |
|  | Gender | 0.09 | [-0.02, 0.19] | -0.13 | [-0.48, 0.20] | -0.03 | [-0.12, 0.05] | **0.10** | **[0.02, 0.18]** | **0.10** | **[0.01, 0.19]** |
|  | Race | **-0.09** | **[-0.15, -0.03]** | -0.13 | [-0.32, 0.06] | **-0.19** | **[-0.24, -0.14]** | **-0.15** | **[-0.20, -0.10]** | **-0.11** | **[-0.16, -0.06]** |
|  | Mom age | 0.00 | [-0.01, 0.02] | -0.02 | [-0.09, 0.04] | **0.04** | **[0.03, 0.05]** | **0.02** | **[0.01, 0.03]** | **0.03** | **[0.02, 0.04]** |
|  | Gestation time | 0.00 | [-0.03, 0.03] | 0.02 | [-0.09, 0.13] | 0.01 | [-0.02, 0.03] | 0.00 | [-0.02, 0.02] | 0.00 | [-0.03, 0.02] |
|  | Birth length | 0.00 | [-0.06, 0.06] | 0.21 | [0.00, 0.41] | 0.01 | [-0.04, 0.06] | 0.01 | [-0.04, 0.06] | 0.00 | [-0.05, 0.05] |
|  | Birth weight | 0.01 | [-0.06, 0.07] | -0.16 | [-0.40, 0.09] | 0.05 | [0.00, 0.11] | 0.05 | [0.00, 0.10] | **0.07** | **[0.01, 0.12]** |
|  | Breastfed | 0.10 | [0.00, 0.21] | 0.17 | [-0.15, 0.49] | **0.20** | **[0.12, 0.28]** | **0.23** | **[0.14, 0.31]** | **0.20** | **[0.12, 0.29]** |
|  | Substance use | -0.01 | [-0.11, 0.09] | 0.13 | [-0.18, 0.46] | **0.11** | **[0.03, 0.20]** | 0.06 | [-0.02, 0.13] | 0.02 | [-0.06, 0.11] |
|  | Mom edu | **0.05** | **[0.03, 0.07]** | 0.02 | [-0.05, 0.09] | **0.07** | **[0.06, 0.09]** | **0.07** | **[0.05, 0.08]** | **0.06** | **[0.04, 0.08]** |
| *Note*. *b* = regression coefficient. *CI* = 95% credible interval. E = extraversion. A = agreeableness. C = conscientiousness. N = neuroticism. O = openness. Pos. Affect = positive affect. Mom age = mom age at birth of child. Gestation time = weeks that mother was pregnant with child. Substance use = if mother reported any substance use during pregnancy. Mom edu = highest education level achieved by mother. Bold values indicate that the credible intervals do not contain 0.00 (for regression coefficients). Results are from models with covariates. All traits are standardized. | | | | | | | | | | | |

| **Table S12a** | | | | | | | | | | | |
| --- | --- | --- | --- | --- | --- | --- | --- | --- | --- | --- | --- |
| *Individual Estimates from the Childhood Temperament-Only Models with Covariates for Relationship and Family Outcomes* | | | | | | | | | | | |
|  |  | Ever Married | | Ever Divorced | | Times Married | | Relationship Satisfaction | | Ever Had Children | |
| Predictor | | *OR* | *CI* | *OR* | *CI* | *IRR* | *CI* | *b* | *CI* | *OR* | *CI* |
| Temperament | | | | | | | | | | | |
|  | Activity | 1.03 | [0.90, 1.18] | 1.04 | [0.73, 1.46] | 1.03 | [0.92, 1.16] | -0.01 | [-0.13, 0.12] | 1.12 | [0.99, 1.28] |
|  | Compliance | **1.19** | **[1.03, 1.38]** | 0.77 | [0.55, 1.09] | 1.12 | [1.00, 1.26] | **0.21** | **[0.07, 0.34]** | 1.08 | [0.94, 1.24] |
|  | Fearful | 0.86 | [0.73, 1.00] | **0.64** | **[0.40, 0.98]** | 0.89 | [0.78, 1.01] | -0.06 | [-0.20, 0.07] | 1.02 | [0.88, 1.18] |
|  | Insecure | 0.95 | [0.82, 1.10] | 0.75 | [0.51, 1.09] | 0.96 | [0.85, 1.09] | -0.03 | [-0.16, 0.10] | 1.09 | [0.95, 1.25] |
|  | Pos. Affect | 0.92 | [0.82, 1.03] | 0.88 | [0.69, 1.13] | 0.93 | [0.85, 1.02] | 0.02 | [-0.09, 0.12] | 0.95 | [0.85, 1.06] |
|  | Predictability | 1.12 | [0.98, 1.29] | 1.10 | [0.79, 1.54] | 1.09 | [0.97, 1.22] | 0.06 | [-0.06, 0.19] | 0.89 | [0.78, 1.02] |
|  | Sociability | 1.10 | [0.95, 1.28] | 0.98 | [0.70, 1.39] | 1.05 | [0.93, 1.18] | 0.06 | [-0.08, 0.19] | 0.93 | [0.80, 1.07] |
| Covariates | | | | | | | | | | | |
|  | Age | **1.25** | **[1.18, 1.32]** | 1.07 | [0.91, 1.26] | **1.18** | **[1.13, 1.24]** | **0.05** | **[0.01, 0.09]** | **1.19** | **[1.13, 1.25]** |
|  | Gender | **1.51** | **[1.16, 1.95]** | 1.41 | [0.71, 2.73] | **1.34** | **[1.08, 1.66]** | 0.02 | [-0.22, 0.27] | **1.93** | **[1.50, 2.50]** |
|  | Race | **0.78** | **[0.66, 0.92]** | 1.29 | [0.89, 1.84] | **0.85** | **[0.73, 0.98]** | -0.14 | [-0.29, 0.00] | **1.31** | **[1.12, 1.51]** |
|  | Mom age | 0.95 | [0.90, 1.01] | 0.91 | [0.79, 1.04] | 0.96 | [0.92, 1.01] | **0.04** | **[0.01, 0.08]** | **0.91** | **[0.86, 0.95]** |
|  | Gestation time | 0.97 | [0.90, 1.05] | 1.04 | [0.86, 1.27] | 0.98 | [0.92, 1.05] | -0.01 | [-0.08, 0.07] | 1.06 | [0.98, 1.14] |
|  | Birth length | 1.08 | [0.91, 1.28] | 1.30 | [0.85, 2.00] | 1.06 | [0.93, 1.22] | 0.11 | [-0.04, 0.25] | 0.91 | [0.78, 1.07] |
|  | Birth weight | 1.16 | [0.98, 1.39] | 0.72 | [0.46, 1.11] | 1.11 | [0.97, 1.28] | -0.06 | [-0.22, 0.10] | 0.90 | [0.76, 1.07] |
|  | Breastfed | 0.92 | [0.71, 1.21] | 0.90 | [0.47, 1.73] | 0.98 | [0.78, 1.21] | 0.12 | [-0.12, 0.36] | **0.59** | **[0.46, 0.76]** |
|  | Substance use | 1.21 | [0.93, 1.58] | 0.78 | [0.41, 1.51] | 1.08 | [0.88, 1.34] | -0.11 | [-0.36, 0.13] | 0.79 | [0.62, 1.02] |
|  | Mom edu | 0.98 | [0.92, 1.04] | 0.99 | [0.85, 1.14] | 0.99 | [0.94, 1.04] | 0.02 | [-0.03, 0.07] | **0.90** | **[0.85, 0.95]** |
| *Note*. *OR* = odds ratio. *b* = regression coefficient. *CI* = 95% credible interval. Pos. Affect = positive affect. Mom age = mom age at birth of child. Gestation time = weeks that mother was pregnant with child. Substance use = if mother reported any substance use during pregnancy. Mom edu = highest education level achieved by mother. Bold values indicate that the credible intervals either do not contain 1.00 (for *OR*s) or do not contain 0.00 (for regression coefficients). Results are from models with covariates. All traits are standardized. | | | | | | | | | | | |

| **Table S12b** | | | | | | | | | | | |
| --- | --- | --- | --- | --- | --- | --- | --- | --- | --- | --- | --- |
| *Individual Estimates from the Adult-Based Personality-Only Models with Covariates for Relationship and Family Outcome* | | | | | | | | | | | |
|  |  | Ever Married | | Ever Divorced | | Times Married | | Relationship Satisfaction | | Ever Had Children | |
| Predictor | | *OR* | *CI* | *OR* | *CI* | *IRR* | *CI* | *b* | *CI* | *OR* | *CI* |
| Personality | | | | | | | | | |  |  |
|  | E | **1.16** | **[1.09, 1.24]** | 1.09 | [0.97, 1.23] | **1.09** | **[1.04, 1.14]** | **0.13** | **[0.07, 0.20]** | 1.06 | [1.00, 1.13] |
|  | A | **1.16** | **[1.08, 1.24]** | 1.01 | [0.89, 1.14] | **1.07** | **[1.02, 1.13]** | 0.07 | [0.00, 0.14] | 0.93 | [0.87, 1.00] |
|  | C | **1.15** | **[1.07, 1.23]** | 1.09 | [0.95, 1.26] | **1.10** | **[1.04, 1.15]** | **0.14** | **[0.07, 0.20]** | **1.16** | **[1.08, 1.24]** |
|  | N | 0.96 | [0.89, 1.02] | **1.14** | **[1.01, 1.30]** | 0.98 | [0.94, 1.03] | **-0.17** | **[-0.24, -0.11]** | 1.02 | [0.95, 1.09] |
|  | O | **0.82** | **[0.77, 0.88]** | **1.14** | **[1.01, 1.29]** | 0.91 | [0.87, 0.95] | -0.02 | [-0.09, 0.04] | **0.92** | **[0.86, 0.99]** |
| Covariates | | | | | | | | | | | |
|  | Age | **1.17** | **[1.14, 1.20]** | **1.05** | **[1.01, 1.10]** | **1.10** | **[1.08, 1.11]** | 0.01 | [-0.01, 0.03] | **1.16** | **[1.13, 1.18]** |
|  | Gender | **1.37** | **[1.19, 1.55]** | 1.10 | [0.86, 1.40] | **1.19** | **[1.09, 1.31]** | 0.05 | [-0.08, 0.19] | **1.92** | **[1.67, 2.20]** |
|  | Race | **0.85** | **[0.79, 0.92]** | 1.15 | [1.00, 1.32] | 0.94 | [0.89, 0.99] | **-0.10** | **[-0.18, -0.02]** | **1.30** | **[1.20, 1.41]** |
|  | Mom age | **0.93** | **[0.91, 0.96]** | **0.92** | **[0.89, 0.96]** | 0.95 | [0.94, 0.97] | **0.04** | **[0.02, 0.06]** | **0.90** | **[0.88, 0.92]** |
|  | Gestation time | 0.98 | [0.95, 1.01] | 1.01 | [0.95, 1.07] | 0.99 | [0.97, 1.01] | 0.01 | [-0.02, 0.05] | 1.02 | [0.99, 1.06] |
|  | Birth length | **1.08** | **[1.01, 1.16]** | 1.09 | [0.96, 1.24] | 1.04 | [0.99, 1.10] | 0.00 | [-0.07, 0.07] | 0.97 | [0.90, 1.04] |
|  | Birth weight | **1.13** | **[1.04, 1.21]** | 1.03 | [0.90, 1.19] | **1.08** | **[1.02, 1.14]** | 0.01 | [-0.07, 0.09] | 0.95 | [0.88, 1.03] |
|  | Breastfed | **1.40** | **[1.23, 1.59]** | 0.99 | [0.80, 1.24] | **1.23** | **[1.12, 1.34]** | 0.11 | [-0.02, 0.24] | **0.72** | **[0.64, 0.82]** |
|  | Substance use | 1.07 | [0.95, 1.21] | 1.12 | [0.89, 1.42] | 1.06 | [0.97, 1.16] | -0.10 | [-0.23, 0.02] | 1.03 | [0.90, 1.16] |
|  | Mom edu | 0.97 | [0.95, 1.00] | 0.97 | [0.93, 1.01] | 0.99 | [0.97, 1.01] | 0.01 | [-0.01, 0.04] | **0.91** | **[0.89, 0.93]** |
| *Note*. *OR* = odds ratio. *IRR* = incidence rate ratio. *b* = regression coefficient. *CI* = 95% credible interval. E = extraversion. A = agreeableness. C = conscientiousness. N = neuroticism. O = openness. Mom age = mom age at birth of child. Gestation time = weeks that mother was pregnant with child. Substance use = if mother reported any substance use during pregnancy. Mom edu = highest education level achieved by mother. Bold values indicate that the credible intervals either do not contain 1.00 (for *OR*s and *IRR*s) or do not contain 0.00 (for regression coefficients). Results are from models with covariates. All traits are standardized. | | | | | | | | | | | |

| **Table S12c** | | | | | | | | | | | |
| --- | --- | --- | --- | --- | --- | --- | --- | --- | --- | --- | --- |
| *Individual Estimates from the Combined Models with Covariates for Relationship and Family Outcomes* | | | | | | | | | | | |
|  |  | Ever Married | | Ever Divorced | | Times Married | | Relationship Satisfaction | | Ever Had Children | |
| Predictor | | *OR* | *CI* | *OR* | *CI* | *IRR* | *CI* | *b* | *CI* | *OR* | *CI* |
| Temperament | | | | | | | | | | | |
|  | Activity | 1.05 | [0.91, 1.21] | 1.03 | [0.72, 1.46] | 1.04 | [0.93, 1.16] | 0.01 | [-0.12, 0.14] | 1.11 | [0.98, 1.27] |
|  | Compliance | **1.19** | **[1.03, 1.39]** | 0.78 | [0.54, 1.12] | 1.12 | [0.99, 1.26] | **0.19** | **[0.05, 0.33]** | 1.07 | [0.93, 1.22] |
|  | Fearful | 0.85 | [0.73, 1.00] | 0.63 | [0.38, 1.01] | 0.89 | [0.77, 1.01] | -0.05 | [-0.19, 0.09] | 1.02 | [0.89, 1.18] |
|  | Insecure | 0.96 | [0.82, 1.12] | 0.74 | [0.50, 1.09] | 0.97 | [0.85, 1.09] | -0.06 | [-0.19, 0.08] | 1.07 | [0.93, 1.23] |
|  | Pos. Affect | 0.92 | [0.81, 1.03] | 0.88 | [0.68, 1.13] | 0.93 | [0.85, 1.01] | 0.03 | [-0.09, 0.13] | 0.95 | [0.85, 1.06] |
|  | Predictability | 1.10 | [0.95, 1.26] | 1.11 | [0.78, 1.60] | 1.08 | [0.96, 1.21] | 0.05 | [-0.08, 0.18] | 0.90 | [0.79, 1.02] |
|  | Sociability | 1.10 | [0.96, 1.27] | 1.00 | [0.71, 1.41] | 1.05 | [0.93, 1.18] | 0.08 | [-0.05, 0.22] | 0.93 | [0.81, 1.06] |
| Personality | | | | | | | | | | | |
|  | E | 1.04 | [0.90, 1.20] | 1.08 | [0.77, 1.55] | 1.02 | [0.91, 1.15] | 0.11 | [-0.02, 0.24] | 1.01 | [0.89, 1.16] |
|  | A | **1.34** | **[1.14, 1.57]** | 0.83 | [0.55, 1.25] | **1.21** | **[1.07, 1.38]** | 0.10 | [-0.03, 0.23] | 0.94 | [0.81, 1.08] |
|  | C | 1.17 | [1.00, 1.38] | 1.07 | [0.73, 1.59] | 1.12 | [0.99, 1.27] | **0.19** | **[0.05, 0.32]** | 1.11 | [0.97, 1.28] |
|  | N | 0.94 | [0.80, 1.10] | 1.14 | [0.76, 1.74] | 0.98 | [0.87, 1.11] | -0.07 | [-0.20, 0.07] | 1.07 | [0.93, 1.24] |
|  | O | **0.84** | **[0.72, 0.98]** | 1.16 | [0.79, 1.72] | 0.89 | [0.79, 1.01] | 0.02 | [-0.12, 0.15] | 0.93 | [0.80, 1.07] |
| Covariates | | | | | | | | | | | |
|  | Age | **1.25** | **[1.18, 1.32]** | 1.07 | [0.90, 1.28] | **1.18** | **[1.13, 1.24]** | **0.07** | **[0.02, 0.11]** | **1.19** | **[1.13, 1.25]** |
|  | Gender | 1.34 | [1.00, 1.80] | 1.43 | [0.70, 2.99] | 1.22 | [0.97, 1.54] | -0.05 | [-0.32, 0.22] | **1.94** | **[1.47, 2.56]** |
|  | Race | **0.79** | **[0.66, 0.94]** | 1.26 | [0.86, 1.84] | **0.85** | **[0.74, 0.99]** | -0.12 | [-0.28, 0.04] | **1.30** | **[1.11, 1.52]** |
|  | Mom age | 0.96 | [0.91, 1.01] | 0.90 | [0.78, 1.04] | 0.97 | [0.93, 1.01] | **0.07** | **[0.02, 0.11]** | **0.90** | **[0.86, 0.95]** |
|  | Gestation time | 0.97 | [0.89, 1.06] | 1.04 | [0.85, 1.28] | 0.98 | [0.92, 1.05] | -0.03 | [-0.11, 0.05] | 1.05 | [0.97, 1.13] |
|  | Birth length | 1.07 | [0.90, 1.27] | 1.33 | [0.86, 2.06] | 1.05 | [0.91, 1.21] | 0.11 | [-0.04, 0.27] | 0.91 | [0.78, 1.06] |
|  | Birth weight | 1.19 | [0.99, 1.43] | 0.71 | [0.45, 1.08] | 1.13 | [0.98, 1.31] | -0.04 | [-0.20, 0.13] | 0.92 | [0.78, 1.08] |
|  | Breastfed | 0.91 | [0.69, 1.20] | 0.95 | [0.48, 1.81] | 0.97 | [0.77, 1.20] | 0.15 | [-0.12, 0.42] | **0.60** | **[0.47, 0.79]** |
|  | Substance use | 1.31 | [1.00, 1.73] | 0.72 | [0.35, 1.47] | 1.13 | [0.90, 1.41] | -0.06 | [-0.33, 0.21] | 0.79 | [0.61, 1.02] |
|  | Mom edu | 0.97 | [0.92, 1.03] | 0.98 | [0.84, 1.14] | 0.99 | [0.94, 1.04] | 0.00 | [-0.05, 0.06] | **0.90** | **[0.85, 0.95]** |
| *Note*. *OR* = odds ratio. *IRR* = incidence rate ratio. *b* = regression coefficient. *CI* = 95% credible interval. E = extraversion. A = agreeableness. C = conscientiousness. N = neuroticism. O = openness. Pos. Affect = positive affect. Mom age = mom age at birth of child. Gestation time = weeks that mother was pregnant with child. Substance use = if mother reported any substance use during pregnancy. Mom edu = highest education level achieved by mother. Bold values indicate that the credible intervals either do not contain 1.00 (for *OR*s and *IRR*s) or do not contain 0.00 (for regression coefficients). Results are from models with covariates. All traits are standardized. | | | | | | | | | | | |

| **Table S13a** | | | | | | | | | |
| --- | --- | --- | --- | --- | --- | --- | --- | --- | --- |
| *Individual Estimates from the Childhood Temperament-Only Models with Covariates for Education, Career, and Financial Outcomes* | | | | | | | | | |
|  |  | Highest Degree | | Employed | | Ever Receive Welfare | | Annual Salary | |
| Predictor | | *b* | *CI* | *OR* | *CI* | *OR* | *CI* | *b* | *CI* |
| Temperament | | | | | | | | | |
|  | Activity | 0.06 | [-0.04, 0.15] | 0.96 | [0.84, 1.08] | 1.08 | [0.85, 1.37] | -0.01 | [-0.04, 0.01] |
|  | Compliance | 0.10 | [-0.00, 0.20] | 1.10 | [0.96, 1.26] | 0.79 | [0.62, 1.02] | 0.00 | [-0.03, 0.03] |
|  | Fearful | -0.10 | [-0.21, 0.01] | 0.93 | [0.80, 1.06] | 1.08 | [0.85, 1.38] | -0.02 | [-0.05, 0.01] |
|  | Insecure | -0.03 | [-0.14, 0.08] | 0.91 | [0.80, 1.04] | **1.34** | **[1.05, 1.71]** | -0.02 | [-0.05, 0.01] |
|  | Pos. Affect | -0.05 | [-0.13, 0.04] | 1.02 | [0.92, 1.13] | 0.90 | [0.75, 1.09] | **-0.03** | **[-0.05, -0.01]** |
|  | Predictability | **0.17** | **[0.07, 0.28]** | 1.08 | [0.96, 1.22] | 0.98 | [0.78, 1.23] | **0.05** | **[0.03, 0.08]** |
|  | Sociability | **0.17** | **[0.06, 0.27]** | 1.12 | [0.98, 1.27] | **0.76** | **[0.60, 0.96]** | **0.05** | **[0.02, 0.08]** |
| Covariates | | | | | | | | | |
|  | Age | **0.21** | **[0.17, 0.25]** | **1.20** | **[1.14, 1.26]** | **1.16** | **[1.05, 1.28]** | **0.04** | **[0.03, 0.05]** |
|  | Gender | **0.60** | **[0.41, 0.80]** | 1.25 | [0.98, 1.60] | 1.28 | [0.82, 2.02] | **-0.06** | **[-0.12, -0.01]** |
|  | Race | **-0.26** | **[-0.38, -0.14]** | **0.81** | **[0.70, 0.94]** | 1.30 | [0.98, 1.73] | **-0.07** | **[-0.10, -0.03]** |
|  | Mom age | **0.08** | **[0.04, 0.11]** | **1.05** | **[1.01, 1.10]** | 1.09 | [1.00, 1.19] | 0.00 | [-0.01, 0.01] |
|  | Gestation time | -0.06 | [-0.12, -0.00] | 1.00 | [0.93, 1.08] | **1.21** | **[1.05, 1.41]** | 0.01 | [-0.01, 0.02] |
|  | Birth length | 0.07 | [-0.04, 0.19] | 0.87 | [0.75, 1.01] | 1.02 | [0.78, 1.34] | 0.00 | [-0.03, 0.03] |
|  | Birth weight | **0.19** | **[0.07, 0.32]** | 1.04 | [0.89, 1.22] | **0.59** | **[0.42, 0.80]** | 0.01 | [-0.03, 0.04] |
|  | Breastfed | 0.18 | [-0.02, 0.37] | 1.00 | [0.78, 1.29] | 0.73 | [0.44, 1.19] | 0.00 | [-0.05, 0.05] |
|  | Substance use | -0.03 | [-0.21, 0.17] | 0.99 | [0.78, 1.26] | 1.32 | [0.83, 2.17] | 0.00 | [-0.05, 0.05] |
|  | Mom edu | **0.25** | **[0.21, 0.29]** | 1.06 | [1.00, 1.11] | 0.94 | [0.86, 1.03] | -0.01 | [-0.02, 0.00] |
| *Note*. *OR* = odds ratio. *b* = regression coefficient. *CI* = 95% credible interval. Pos. Affect = positive affect. Mom age = mom age at birth of child. Gestation time = weeks that mother was pregnant with child. Substance use = if mother reported any substance use during pregnancy. Mom edu = highest education level achieved by mother. Bold values indicate that the credible intervals either do not contain 1.00 (for *OR*s) or do not contain 0.00 (for regression coefficients). Results are from models with covariates. All traits are standardized. | | | | | | | | | |

| **Table S13b** | | | | | | | | | |
| --- | --- | --- | --- | --- | --- | --- | --- | --- | --- |
| *Individual Estimates from the Adult-Based Personality-Only Models with Covariates for Education, Career, and Financial Outcomes* | | | | | | | | | |
|  |  | Highest Degree | | Employed | | Ever Receive Welfare | | Annual Salary | |
| Predictor | | *b* | *CI* | *OR* | *CI* | *OR* | *CI* | *b* | *CI* |
| Personality | | | | | | | | | |
|  | E | **0.11** | **[0.07, 0.16]** | **1.14** | **[1.07, 1.22]** | **0.80** | **[0.72, 0.88]** | **0.10** | **[0.08, 0.12]** |
|  | A | 0.00 | [-0.06, 0.05] | 0.97 | [0.91, 1.04] | **1.15** | **[1.04, 1.28]** | **-0.05** | **[-0.07, -0.03]** |
|  | C | **0.11** | **[0.06, 0.17]** | **1.08** | **[1.02, 1.16]** | **0.87** | **[0.79, 0.97]** | **0.04** | **[0.02, 0.06]** |
|  | N | **-0.21** | **[-0.27, -0.16]** | **0.89** | **[0.84, 0.96]** | **1.24** | **[1.12, 1.37]** | **-0.03** | **[-0.05, -0.01]** |
|  | O | -0.05 | [-0.10, -0.00] | 1.05 | [0.98, 1.12] | **0.88** | **[0.79, 0.97]** | -0.01 | [-0.03, 0.01] |
| Covariates | | | | | | | | | |
|  | Age | **0.12** | **[0.10, 0.14]** | **1.12** | **[1.09, 1.15]** | **1.08** | **[1.05, 1.13]** | **0.05** | **[0.04, 0.06]** |
|  | Gender | **0.64** | **[0.53, 0.75]** | 1.04 | [0.91, 1.18] | **1.32** | **[1.07, 1.62]** | **-0.13** | **[-0.17, -0.08]** |
|  | Race | **-0.21** | **[-0.28, -0.15]** | **0.83** | **[0.77, 0.90]** | **1.33** | **[1.18, 1.50]** | **-0.07** | **[-0.09, -0.04]** |
|  | Mom age | **0.07** | **[0.05, 0.08]** | **1.06** | **[1.03, 1.08]** | 0.98 | [0.95, 1.02] | -0.01 | [-0.01, -0.00] |
|  | Gestation time | -0.02 | [-0.05, 0.00] | 0.98 | [0.94, 1.01] | 1.04 | [0.98, 1.09] | 0.00 | [-0.02, 0.01] |
|  | Birth length | 0.04 | [-0.01, 0.10] | 0.99 | [0.92, 1.06] | **0.89** | **[0.80, 0.98]** | **0.03** | **[0.01, 0.06]** |
|  | Birth weight | **0.09** | **[0.02, 0.15]** | 1.05 | [0.97, 1.13] | **0.86** | **[0.76, 0.96]** | 0.02 | [-0.01, 0.04] |
|  | Breastfed | **0.31** | **[0.21, 0.41]** | 1.13 | [0.98, 1.28] | **0.62** | **[0.51, 0.77]** | **0.08** | **[0.04, 0.12]** |
|  | Substance use | -0.02 | [-0.12, 0.08] | 0.99 | [0.87, 1.12] | **1.43** | **[1.18, 1.73]** | -0.01 | [-0.04, 0.03] |
|  | Mom edu | **0.22** | **[0.20, 0.24]** | **1.05** | **[1.02, 1.07]** | **0.93** | **[0.90, 0.97]** | 0.00 | [-0.01, 0.01] |
| *Note*. *OR* = odds ratio. *b* = regression coefficient. *CI* = 95% credible interval. E = extraversion. A = agreeableness. C = conscientiousness. N = neuroticism. O = openness. Mom age = mom age at birth of child. Gestation time = weeks that mother was pregnant with child. Substance use = if mother reported any substance use during pregnancy. Mom edu = highest education level achieved by mother. Bold values indicate that the credible intervals either do not contain 1.00 (for *OR*s) or do not contain 0.00 (for regression coefficients). Results are from models with covariates. All traits are standardized. | | | | | | | | | |

| **Table S13c** | | | | | | | | | |
| --- | --- | --- | --- | --- | --- | --- | --- | --- | --- |
| *Individual Estimates from the Combined Models with Covariates for Education, Career, and Financial Outcomes* | | | | | | | | | |
|  |  | Highest Degree | | Employed | | Ever Receive Welfare | | Annual Salary | |
| Predictor | | *b* | *CI* | *OR* | *CI* | *OR* | *CI* | *b* | *CI* |
| Temperament | | | | | | | | | |
|  | Activity | 0.07 | [-0.03, 0.17] | 0.97 | [0.85, 1.10] | 1.03 | [0.81, 1.32] | -0.01 | [-0.04, 0.02] |
|  | Compliance | 0.08 | [-0.03, 0.19] | 1.08 | [0.95, 1.24] | 0.77 | [0.60, 1.00] | 0.00 | [-0.03, 0.03] |
|  | Fearful | -0.09 | [-0.20, 0.01] | 0.93 | [0.80, 1.07] | 1.07 | [0.84, 1.35] | -0.01 | [-0.04, 0.02] |
|  | Insecure | -0.02 | [-0.13, 0.09] | 0.91 | [0.80, 1.05] | **1.28** | **[1.01, 1.63]** | -0.02 | [-0.05, 0.01] |
|  | Pos. Affect | -0.05 | [-0.14, 0.03] | 1.00 | [0.90, 1.11] | 0.92 | [0.77, 1.11] | **-0.04** | **[-0.06, -0.01]** |
|  | Predictability | **0.15** | **[0.05, 0.26]** | 1.07 | [0.95, 1.22] | 1.02 | [0.81, 1.29] | **0.05** | **[0.02, 0.07]** |
|  | Sociability | **0.17** | **[0.06, 0.27]** | 1.12 | [0.98, 1.28] | 0.77 | [0.60, 1.00] | **0.05** | **[0.02, 0.08]** |
| Personality | | | | | | | | | |
|  | E | 0.06 | [-0.04, 0.16] | 1.12 | [0.98, 1.27] | **0.75** | **[0.58, 0.96]** | **0.06** | **[0.04, 0.09]** |
|  | A | 0.07 | [-0.04, 0.18] | 0.94 | [0.82, 1.07] | 1.00 | [0.77, 1.29] | **-0.03** | **[-0.06, -0.01]** |
|  | C | **0.12** | **[0.01, 0.23]** | 1.04 | [0.92, 1.19] | 0.92 | [0.72, 1.17] | 0.02 | [-0.01, 0.04] |
|  | N | **-0.28** | **[-0.38, -0.17]** | 0.93 | [0.81, 1.06] | **1.49** | **[1.15, 1.92]** | -0.01 | [-0.04, 0.02] |
|  | O | -0.10 | [-0.20, 0.00] | 1.09 | [0.96, 1.25] | 0.85 | [0.66, 1.10] | 0.01 | [-0.02, 0.04] |
| Covariates | | | | | | | | | |
|  | Age | **0.21** | **[0.17, 0.25]** | **1.19** | **[1.12, 1.25]** | **1.18** | **[1.06, 1.30]** | **0.04** | **[0.03, 0.05]** |
|  | Gender | **0.69** | **[0.48, 0.90]** | 1.30 | [1.00, 1.71] | 1.22 | [0.74, 2.00] | -0.05 | [-0.10, 0.01] |
|  | Race | **-0.25** | **[-0.37, -0.13]** | **0.79** | **[0.68, 0.91]** | 1.34 | [0.99, 1.79] | **-0.07** | **[-0.10, -0.03]** |
|  | Mom age | **0.08** | **[0.04, 0.11]** | 1.04 | [0.99, 1.09] | 1.10 | [1.00, 1.21] | 0.00 | [-0.01, 0.01] |
|  | Gestation time | **-0.06** | **[-0.12, -0.01]** | 1.00 | [0.93, 1.08] | **1.22** | **[1.05, 1.41]** | 0.00 | [-0.01, 0.02] |
|  | Birth length | 0.06 | [-0.06, 0.18] | 0.85 | [0.73, 1.00] | 1.06 | [0.81, 1.41] | -0.01 | [-0.04, 0.03] |
|  | Birth weight | **0.21** | **[0.08, 0.33]** | 1.05 | [0.89, 1.23] | **0.58** | **[0.43, 0.81]** | 0.01 | [-0.03, 0.05] |
|  | Breastfed | 0.16 | [-0.04, 0.36] | 1.03 | [0.79, 1.32] | 0.74 | [0.45, 1.21] | 0.01 | [-0.04, 0.06] |
|  | Substance use | 0.04 | [-0.15, 0.23] | 0.99 | [0.77, 1.27] | 1.23 | [0.76, 2.01] | 0.00 | [-0.05, 0.06] |
|  | Mom edu | **0.25** | **[0.21, 0.29]** | 1.05 | [1.00, 1.10] | 0.96 | [0.88, 1.06] | -0.01 | [-0.02, 0.00] |
| *Note*. *OR* = odds ratio. *b* = regression coefficient. *CI* = 95% credible interval. E = extraversion. A = agreeableness. C = conscientiousness. N = neuroticism. O = openness. Pos. Affect = positive affect. Mom age = mom age at birth of child. Gestation time = weeks that mother was pregnant with child. Substance use = if mother reported any substance use during pregnancy. Mom edu = highest education level achieved by mother. Bold values indicate that the credible intervals either do not contain 1.00 (for *OR*s) or do not contain 0.00 (for regression coefficients). Results are from models with covariates. All traits are standardized. | | | | | | | | | |

| **Table S14a** | | | | | |
| --- | --- | --- | --- | --- | --- |
| *Individual Estimates from the Childhood Temperament-Only Models with Covariates for Civic Engagement Outcomes* | | | | | |
|  |  | Ever Volunteer | | Religious | |
| Predictor | | *OR* | *CI* | *OR* | *CI* |
| Temperament | | | | | |
|  | Activity | 0.96 | [0.84, 1.11] | 0.85 | [0.62, 1.16] |
|  | Compliance | 1.07 | [0.93, 1.24] | 0.89 | [0.62, 1.27] |
|  | Fearful | 0.94 | [0.82, 1.09] | 1.28 | [0.88, 1.87] |
|  | Insecure | 0.90 | [0.78, 1.04] | 1.20 | [0.85, 1.70] |
|  | Pos. Affect | 1.04 | [0.93, 1.17] | 0.94 | [0.70, 1.23] |
|  | Predictability | 1.04 | [0.91, 1.18] | 1.18 | [0.85, 1.62] |
|  | Sociability | **1.28** | **[1.11, 1.47]** | 0.97 | [0.68, 1.37] |
| Covariates | | | | | |
|  | Age | 1.04 | [0.99, 1.09] | 1.07 | [0.95, 1.20] |
|  | Gender | **1.57** | **[1.20, 2.07]** | 1.17 | [0.62, 2.24] |
|  | Race | 0.93 | [0.79, 1.09] | 0.98 | [0.68, 1.45] |
|  | Mom age | 1.04 | [0.99, 1.09] | 1.00 | [0.89, 1.12] |
|  | Gestation time | 1.03 | [0.95, 1.11] | 0.87 | [0.71, 1.06] |
|  | Birth length | 1.11 | [0.95, 1.29] | 1.09 | [0.75, 1.55] |
|  | Birth weight | 0.93 | [0.79, 1.10] | 1.22 | [0.82, 1.86] |
|  | Breastfed | 1.16 | [0.88, 1.54] | 1.28 | [0.65, 2.47] |
|  | Substance use | 0.89 | [0.69, 1.15] | 0.65 | [0.33, 1.23] |
|  | Mom edu | **1.11** | **[1.05, 1.18]** | 1.08 | [0.95, 1.23] |
| *Note*. *OR* = odds ratio. *CI* = 95% credible interval. E = extraversion. A = agreeableness. C = conscientiousness. N = neuroticism. O = openness. Mom age = mom age at birth of child. Gestation time = weeks that mother was pregnant with child. Substance use = if mother reported any substance use during pregnancy. Mom edu = highest education level achieved by mother. Bold values indicate that the credible intervals do not contain 1.00. Results are from models with covariates. All traits are standardized. | | | | | |

| **Table S14b** | | | | | |
| --- | --- | --- | --- | --- | --- |
| *Individual Estimates from the Adult-Based Personality-Only Models with Covariates for Civic Engagement Outcomes* | | | | | |
|  |  | Ever Volunteer | | Religious | |
| Predictor | | *OR* | *CI* | *OR* | *CI* |
| Personality | | | | | |
|  | E | **1.23** | **[1.15, 1.32]** | 1.05 | [0.88, 1.25] |
|  | A | 1.05 | [0.98, 1.13] | 1.16 | [0.96, 1.38] |
|  | C | 1.07 | [1.00, 1.15] | 0.99 | [0.82, 1.18] |
|  | N | **0.91** | **[0.85, 0.98]** | 1.05 | [0.87, 1.27] |
|  | O | 1.06 | [0.99, 1.14] | 0.90 | [0.75, 1.09] |
| Covariates | | | | | |
|  | Age | **1.04** | **[1.02, 1.07]** | **1.07** | **[1.01, 1.14]** |
|  | Gender | **1.45** | **[1.26, 1.67]** | 1.28 | [0.88, 1.86] |
|  | Race | **0.90** | **[0.83, 0.97]** | **1.53** | **[1.20, 1.96]** |
|  | Mom age | **1.05** | **[1.02, 1.07]** | 0.98 | [0.92, 1.04] |
|  | Gestation time | 1.01 | [0.98, 1.05] | 0.95 | [0.85, 1.04] |
|  | Birth length | 0.96 | [0.90, 1.04] | 1.08 | [0.89, 1.31] |
|  | Birth weight | 1.02 | [0.94, 1.10] | 1.06 | [0.86, 1.31] |
|  | Breastfed | **1.29** | **[1.12, 1.47]** | 0.90 | [0.63, 1.28] |
|  | Substance use | 1.01 | [0.89, 1.16] | **0.67** | **[0.46, 0.96]** |
|  | Mom edu | **1.12** | **[1.09, 1.15]** | 1.07 | [1.00, 1.16] |
| *Note*. *OR* = odds ratio. *CI* = 95% credible interval. E = extraversion. A = agreeableness. C = conscientiousness. N = neuroticism. O = openness. Mom age = mom age at birth of child. Gestation time = weeks that mother was pregnant with child. Substance use = if mother reported any substance use during pregnancy. Mom edu = highest education level achieved by mother. Bold values indicate that the credible intervals do not contain 1.00. Results are from models with covariates. All traits are standardized. | | | | | |

| **Table S14c** | | | | | |
| --- | --- | --- | --- | --- | --- |
| *Individual Estimates from the Combined Models with Covariates for Civic Engagement Outcomes* | | | | | |
|  |  | Ever Volunteer | | Religious | |
| Predictor | | *OR* | *CI* | *OR* | *CI* |
| Temperament | | | | | |
|  | Activity | 0.98 | [0.86, 1.13] | 0.85 | [0.62, 1.18] |
|  | Compliance | 1.06 | [0.92, 1.22] | 0.88 | [0.63, 1.24] |
|  | Fearful | 0.94 | [0.81, 1.09] | 1.27 | [0.85, 1.93] |
|  | Insecure | 0.91 | [0.78, 1.06] | 1.24 | [0.88, 1.77] |
|  | Pos. Affect | 1.03 | [0.92, 1.16] | 0.97 | [0.73, 1.25] |
|  | Predictability | 1.01 | [0.88, 1.16] | 1.12 | [0.80, 1.54] |
|  | Sociability | **1.26** | **[1.09, 1.46]** | 0.99 | [0.69, 1.42] |
| Personality | | | | | |
|  | E | 1.15 | [1.00, 1.33] | 1.06 | [0.77, 1.48] |
|  | A | 1.16 | [1.00, 1.35] | **1.43** | **[1.04, 1.98]** |
|  | C | **1.21** | **[1.05, 1.40]** | 1.08 | [0.77, 1.51] |
|  | N | 0.89 | [0.78, 1.03] | 0.93 | [0.69, 1.30] |
|  | O | 1.05 | [0.90, 1.20] | **0.61** | **[0.42, 0.88]** |
| Covariates | | | | | |
|  | Age | 1.04 | [0.99, 1.09] | 1.04 | [0.92, 1.17] |
|  | Gender | **1.51** | **[1.12, 2.01]** | 1.04 | [0.52, 2.12] |
|  | Race | 0.93 | [0.78, 1.09] | 1.00 | [0.68, 1.50] |
|  | Mom age | 1.04 | [0.99, 1.10] | 0.99 | [0.88, 1.11] |
|  | Gestation time | 1.03 | [0.95, 1.11] | 0.89 | [0.73, 1.08] |
|  | Birth length | 1.09 | [0.93, 1.28] | 1.10 | [0.75, 1.57] |
|  | Birth weight | 0.93 | [0.79, 1.11] | 1.23 | [0.82, 1.88] |
|  | Breastfed | 1.19 | [0.91, 1.57] | 1.25 | [0.65, 2.34] |
|  | Substance use | 0.95 | [0.73, 1.25] | 0.69 | [0.33, 1.36] |
|  | Mom edu | **1.10** | **[1.04, 1.17]** | 1.08 | [0.93, 1.24] |
| *Note*. *OR* = odds ratio. *CI* = 95% credible interval. E = extraversion. A = agreeableness. C = conscientiousness. N = neuroticism. O = openness. Mom age = mom age at birth of child. Gestation time = weeks that mother was pregnant with child. Substance use = if mother reported any substance use during pregnancy. Mom edu = highest education level achieved by mother. Bold values indicate that the credible intervals do not contain 1.00. Results are from models with covariates. All traits are standardized. | | | | | |

| **Table S15** | | | | | | | | | | | | | | | |
| --- | --- | --- | --- | --- | --- | --- | --- | --- | --- | --- | --- | --- | --- | --- | --- |
| *Comparison of Estimates from the Temperament-Only Models Without and With Covariates* | | | | | | | | | | | | | | | |
|  |  | Activity | | Compliance | | Fearful | | Insecure | | Pos. Affect | | Predictability | | Sociability | |
| Outcome | | W/O | With | W/O | With | W/O | With | W/O | With | W/O | With | W/O | With | W/O | With |
| Health | |  |  |  |  |  |  |  |  |  |  |  |  |  |  |
|  | Health Status | **-0.09** | -0.07 | **0.13** | **0.13** | -0.03 | -0.04 | **-0.12** | -0.04 | 0.01 | 0.01 | -0.02 | -0.05 | 0.05 | -0.01 |
|  | BMI | 0.03 | 0.03 | **-0.06** | **-0.06** | 0.00 | -0.01 | 0.00 | -0.01 | 0.02 | 0.01 | -0.04 | -0.02 | 0.00 | 0.00 |
| Internalizing | | | | | | | | | | | | | | | |
|  | Anxiety | **1.32** | 1.34 | 1.01 | 0.92 | 1.04 | 1.18 | 0.92 | 0.84 | 1.01 | 0.97 | 1.25 | 1.35 | 1.15 | 1.01 |
|  | Depression | 1.20 | 1.16 | 0.98 | 0.98 | 0.88 | 1.04 | 1.33 | 1.34 | 0.96 | 0.95 | 1.01 | 0.93 | **1.46** | **1.49** |
|  | Counselor | 1.00 | 1.00 | **0.83** | **0.76** | 0.99 | 1.02 | 1.04 | 1.03 | 1.00 | 1.05 | 1.06 | 1.00 | 1.01 | 1.03 |
|  | Suicide | 0.93 | 0.95 | 0.95 | 0.91 | 1.01 | 1.05 | 1.08 | 1.00 | 1.01 | 0.98 | 1.06 | 1.02 | 1.15 | 1.17 |
| Externalizing | | | | | | | | | | | | | | | |
|  | ADHD | 1.02 | 0.91 | **0.67** | **0.58** | 0.87 | 0.76 | 0.78 | 0.79 | 0.90 | 1.01 | 1.13 | 1.14 | 0.81 | 0.87 |
|  | Ever jail | 1.00 | 0.90 | 1.00 | 1.00 | 1.15 | 1.06 | 1.12 | 1.11 | 1.08 | **1.24** | **0.72** | **0.77** | **0.77** | 0.87 |
|  | Num. Substances | 0.99 | 0.98 | 0.99 | 1.00 | 1.01 | 1.00 | **0.96** | **0.96** | 0.99 | 0.99 | 1.01 | 1.02 | 1.01 | 1.02 |
| Cognitive | |  |  |  |  |  |  |  |  |  |  |  |  |  |  |
|  | Digit Span | **-0.08** | **-0.06** | **0.10** | **0.08** | 0.02 | 0.03 | -0.04 | -0.03 | 0.00 | -0.03 | 0.01 | -0.01 | **0.17** | **0.15** |
|  | Word Recall | **-0.16** | **-0.19** | 0.01 | -0.03 | -0.11 | -0.06 | -0.11 | -0.15 | 0.10 | 0.08 | 0.09 | 0.05 | 0.10 | 0.10 |
|  | PIAT Math | **-0.09** | -0.03 | **0.12** | **0.11** | **-0.09** | -0.02 | **-0.07** | -0.01 | -0.02 | 0.02 | **0.09** | 0.03 | **0.24** | **0.16** |
|  | PIAT Read Comp | **-0.05** | -0.02 | **0.14** | **0.12** | **-0.08** | -0.01 | -0.05 | -0.01 | **-0.05** | -0.01 | **0.09** | 0.03 | **0.25** | **0.18** |
|  | PIAT Read Rec | **-0.07** | -0.04 | **0.12** | **0.11** | **-0.07** | -0.02 | -0.04 | -0.01 | **-0.05** | -0.01 | **0.10** | 0.04 | **0.23** | **0.16** |
| Relationships & Family | |  |  |  |  |  |  |  |  |  |  |  |  |  |  |
|  | Ever Married | 1.05 | 1.03 | **1.23** | **1.19** | 0.92 | 0.86 | 0.99 | 0.95 | 0.93 | 0.92 | 1.01 | 1.12 | 1.09 | 1.10 |
|  | Ever Divorced | 1.09 | 1.04 | 0.74 | 0.77 | 0.80 | **0.64** | 0.88 | 0.75 | 0.90 | 0.88 | 1.04 | 1.10 | 0.91 | 0.98 |
|  | Times Married | 1.04 | 1.03 | **1.17** | 1.12 | 0.93 | 0.89 | 0.99 | 0.96 | 0.93 | 0.93 | 1.01 | 1.09 | 1.07 | 1.05 |
|  | Rel. Satisfaction | -0.03 | -0.01 | **0.21** | **0.21** | -0.06 | -0.06 | -0.10 | -0.03 | 0.03 | 0.02 | 0.06 | 0.06 | 0.12 | 0.06 |
|  | Ever Children | **1.18** | 1.12 | 1.05 | 1.08 | 1.12 | 1.02 | **1.21** | 1.09 | 0.97 | 0.95 | **0.78** | 0.89 | **0.84** | 0.93 |
| Education, Career, & Financial | |  |  |  |  |  |  |  |  |  |  |  |  |  |  |
|  | Highest Degree | 0.00 | 0.06 | **0.21** | 0.10 | -0.07 | -0.10 | **-0.11** | -0.03 | -0.06 | -0.05 | **0.16** | **0.17** | **0.21** | **0.17** |
|  | Employed | 0.97 | 0.96 | 1.12 | 1.10 | 0.99 | 0.93 | **0.89** | 0.91 | 0.97 | 1.02 | 1.05 | 1.08 | 1.10 | 1.12 |
|  | Ever Welfare | 1.16 | 1.08 | 0.82 | 0.79 | 1.15 | 1.08 | **1.41** | **1.34** | 0.88 | 0.90 | 0.90 | 0.98 | **0.69** | **0.76** |
|  | Annual Salary | -0.01 | -0.01 | 0.01 | 0.00 | -0.02 | -0.02 | -0.02 | -0.02 | **-0.03** | **-0.03** | 0.02 | **0.05** | **0.03** | **0.05** |
| Civic Engagement | |  |  |  |  |  |  |  |  |  |  |  |  |  |  |
|  | Ever Volunteered | 0.92 | 0.96 | **1.16** | 1.07 | 0.94 | 0.94 | 0.91 | 0.90 | 1.02 | 1.04 | 1.08 | 1.04 | **1.29** | **1.28** |
|  | Religious | 0.93 | 0.85 | 0.90 | 0.89 | 1.31 | 1.28 | 1.18 | 1.20 | 1.00 | 0.94 | 1.10 | 1.18 | 1.10 | 0.97 |
| *Note*. W/O = without covariates. With = with covariates. Bolded values indicate that the credible intervals did not contain 1.00 (for *OR*s and *IRR*s) or 0.00 (for *b*s). See previous supplementary tables for the metric of all estimates per outcome (i.e., *OR*, *IRR*, or *b*). | | | | | | | | | | | | | | | |

| **Table S16** | | | | | | | | | | | |
| --- | --- | --- | --- | --- | --- | --- | --- | --- | --- | --- | --- |
| *Comparison of Estimates from the Personality-Only Models Without and With Covariates* | | | | | | | | | | | |
|  |  | Extraversion | | Agreeableness | | Conscientiousness | | Neuroticism | | Openness | |
| Outcome | | W/O | With | W/O | With | W/O | With | W/O | With | W/O | With |
| Health | |  |  |  |  |  |  |  |  |  |  |
|  | Health Status | **0.15** | **0.13** | 0.00 | 0.00 | **0.16** | **0.24** | **-0.28** | **-0.26** | 0.02 | 0.00 |
|  | BMI | 0.00 | 0.02 | -0.02 | -0.01 | 0.00 | -0.01 | 0.01 | 0.02 | 0.00 | 0.00 |
| Internalizing | | | | | | | | | | | |
|  | Anxiety | 0.88 | 0.87 | **1.24** | 1.19 | **1.15** | **1.23** | **1.63** | **1.52** | **1.21** | 1.18 |
|  | Depression | **0.80** | **0.79** | **1.19** | 1.10 | 1.05 | 1.17 | **1.58** | **1.53** | **1.23** | **1.22** |
|  | Counselor | 1.04 | 1.02 | **1.09** | 1.02 | **0.83** | **0.82** | **1.54** | **1.51** | **1.14** | **1.13** |
|  | Suicide | **0.87** | **0.87** | **1.19** | **1.20** | **0.81** | **0.85** | **2.00** | **2.04** | **1.37** | **1.35** |
| Externalizing | | | | | | | | | | | |
|  | ADHD | 1.10 | 1.17 | 0.94 | 1.04 | **0.72** | **0.85** | 1.08 | **1.30** | 0.98 | 1.00 |
|  | Ever jail | **0.77** | **0.86** | **0.75** | **0.89** | 1.07 | 1.03 | **1.16** | **1.36** | **1.20** | **1.20** |
|  | Num. Substances | **1.04** | **1.06** | **0.97** | 0.98 | **0.94** | **0.93** | **1.07** | **1.09** | **1.07** | **1.07** |
| Cognitive | |  |  |  |  |  |  |  |  |  |  |
|  | Digit Span | **0.10** | **0.07** | **0.03** | -0.01 | **-0.05** | -0.02 | -0.02 | -0.01 | **0.07** | **0.07** |
|  | Word Recall | **0.09** | 0.06 | **0.08** | 0.05 | 0.01 | 0.01 | -0.03 | -0.03 | 0.01 | 0.03 |
|  | PIAT Math | **0.10** | **0.06** | 0.01 | 0.01 | **-0.13** | **-0.06** | **-0.09** | **-0.04** | **0.05** | **0.03** |
|  | PIAT Read Comp | **0.09** | **0.06** | **0.08** | **0.05** | **-0.11** | **-0.06** | **-0.06** | -0.03 | **0.06** | **0.04** |
|  | PIAT Read Rec | **0.08** | **0.05** | **0.07** | **0.05** | **-0.09** | **-0.04** | **-0.05** | -0.02 | **0.07** | **0.06** |
| Relationships & Family | |  |  |  |  |  |  |  |  |  |  |
|  | Ever Married | **1.12** | **1.16** | **1.22** | **1.16** | **1.25** | **1.15** | 1.00 | 0.96 | **0.83** | **0.82** |
|  | Ever Divorced | 1.03 | 1.09 | 1.01 | 1.01 | 1.13 | 1.09 | **1.15** | **1.14** | 1.11 | **1.14** |
|  | Times Married | **1.08** | **1.09** | **1.13** | **1.07** | **1.18** | **1.10** | 1.01 | 0.98 | **0.89** | 0.91 |
|  | Rel. Satisfaction | **0.13** | **0.13** | **0.09** | 0.07 | **0.11** | **0.14** | **-0.19** | **-0.17** | -0.02 | -0.02 |
|  | Ever Children | 1.00 | 1.06 | 1.05 | 0.93 | **1.38** | **1.16** | **1.14** | 1.02 | **0.88** | **0.92** |
| Education, Career, & Financial | |  |  |  |  |  |  |  |  |  |  |
|  | Highest Degree | **0.17** | **0.11** | **0.12** | 0.00 | **0.08** | **0.11** | **-0.16** | **-0.21** | -0.04 | -0.05 |
|  | Employed | **1.15** | **1.14** | 1.01 | 0.97 | **1.09** | **1.08** | **0.89** | **0.89** | 1.04 | 1.05 |
|  | Ever Welfare | **0.77** | **0.80** | **1.21** | **1.15** | 0.98 | **0.87** | **1.34** | **1.24** | **0.83** | **0.88** |
|  | Annual Salary | **0.08** | **0.10** | **-0.03** | **-0.05** | **0.07** | **0.04** | **-0.03** | **-0.03** | -0.01 | -0.01 |
| Civic Engagement | |  |  |  |  |  |  |  |  |  |  |
|  | Ever Volunteered | **1.30** | **1.23** | **1.13** | 1.05 | 1.03 | 1.07 | 0.95 | **0.91** | 1.06 | 1.06 |
|  | Religious | 1.10 | 1.05 | **1.23** | 1.16 | 1.11 | 0.99 | 1.08 | 1.05 | 0.97 | 0.90 |
| *Note*. W/O = without covariates. With = with covariates. Bolded values indicate that the credible intervals did not contain 1.00 (for *OR*s and *IRR*s) or 0.00 (for *b*s). See previous supplementary tables for the metric of all estimates per outcome (i.e., *OR*, *IRR*, or *b*). | | | | | | | | | | | |

| **Table S17** | |  |  |
| --- | --- | --- | --- |
| *Model R^2^ Values from the Personality-Only Models for All Outcomes Using the mini-IPIP* | | | |
| Domain | Outcome | *R^2^* | *CI* |
| Health | |  |  |
|  | Health Status at Last Wave | 5.73 | [3.85, 7.80] |
|  | BMI at Last Wave | 5.10 | [3.58, 6.78] |
| Internalizing | |  |  |
|  | Anxiety | 7.33 | [2.89, 12.58] |
|  | Depression | 12.65 | [6.33, 19.50] |
|  | Counselor | 4.38 | [2.74, 6.23] |
|  | Suicide | 5.82 | [3.44, 8.49] |
| Externalizing | |  |  |
|  | ADHD | 5.94 | [2.31, 10.83] |
|  | Ever jail | 1.84 | [0.68, 3.51] |
|  | Number of Substances | 4.11 | [2.49, 5.94] |
| Cognitive | |  |  |
|  | Digit Span | 5.85 | [3.92, 8.07] |
|  | Word Recall | 5.18 | [1.49, 9.80] |
|  | PIAT Math | 8.17 | [6.01, 10.52] |
|  | PIAT Read Comprehension | 7.88 | [5.63, 10.26] |
|  | PIAT Read Recognition | 7.08 | [4.95, 9.36] |
| Relationship & Family | |  |  |
|  | Ever Married | 2.71 | [1.39, 4.23] |
|  | Ever Divorced | 2.35 | [0.57, 4.96] |
|  | Times Married | 2.68 | [1.20, 4.70] |
|  | Relationship Satisfaction | 1.22 | [0.43, 2.35] |
|  | Ever Have Children | 4.07 | [2.54, 5.82] |
| Education, Career, Financial | |  |  |
|  | Highest Degree | 5.28 | [3.52, 7.20] |
|  | Employed at Last Wave | 2.93 | [1.54, 4.64] |
|  | Annual Salary | 3.05 | [1.44, 5.26] |
|  | Ever Receive Welfare | 1.83 | [1.06, 2.75] |
| Civic Engagement | |  |  |
|  | Religious | 2.19 | [0.57, 4.87] |
|  | Ever Volunteered | 3.34 | [1.85, 5.09] |
| *Note*. R^2^ values are presented as percentages. Results are from models without covariates. | | | |

| **Table S18** | | | | | | | | | | | |
| --- | --- | --- | --- | --- | --- | --- | --- | --- | --- | --- | --- |
| *Comparison of Estimates from the Personality-Only Models When Assessed with the TIPI vs mini-IPIP* | | | | | | | | | | | |
|  |  | Extraversion | | Agreeableness | | Conscientiousness | | Neuroticism | | Openness | |
| Outcome | | TIPI | mini-IPIP | TIPI | mini-IPIP | TIPI | mini-IPIP | TIPI | mini-IPIP | TIPI | mini-IPIP |
| Health | |  |  |  |  |  |  |  |  |  |  |
|  | Health Status | **0.15** | **0.11** | 0.00 | -0.02 | **0.16** | **0.21** | **-0.28** | **-0.30** | 0.02 | 0.09 |
|  | BMI | 0.00 | **0.07** | -0.02 | **-0.05** | 0.00 | 0.00 | 0.01 | 0.03 | 0.00 | -0.03 |
| Internalizing | | | | | | | | | | | |
|  | Anxiety | 0.88 | **0.74** | **1.24** | **1.50** | **1.15** | 1.09 | **1.63** | **1.47** | **1.21** | 1.05 |
|  | Depression | **0.80** | **0.57** | **1.19** | **1.47** | 1.05 | 0.88 | **1.58** | **1.62** | **1.23** | 1.13 |
|  | Counselor | 1.04 | 1.00 | **1.09** | **1.22** | **0.83** | **0.85** | **1.54** | **1.43** | **1.14** | 1.06 |
|  | Suicide | **0.87** | **0.85** | **1.19** | 1.17 | **0.81** | 0.90 | **2.00** | **1.87** | **1.37** | 1.16 |
| Externalizing | | | | | | | | | | | |
|  | ADHD | 1.10 | 1.35 | 0.94 | **0.71** | **0.72** | **0.60** | 1.08 | 1.26 | 0.98 | 1.05 |
|  | Ever jail | **0.77** | 1.07 | **0.75** | **0.68** | 1.07 | 0.99 | **1.16** | 1.18 | **1.20** | 1.09 |
|  | Num. Substances | **1.04** | **1.07** | **0.97** | **0.94** | **0.94** | **0.95** | **1.07** | **1.05** | **1.07** | **1.08** |
| Cognitive | |  |  |  |  |  |  |  |  |  |  |
|  | Digit Span | **0.10** | -0.01 | **0.03** | **0.10** | **-0.05** | -0.02 | -0.02 | **-0.07** | **0.07** | **0.17** |
|  | Word Recall | **0.09** | -0.04 | **0.08** | 0.05 | 0.01 | 0.00 | -0.03 | -0.08 | 0.01 | **0.15** |
|  | PIAT Math | **0.10** | **-0.06** | 0.01 | **0.09** | **-0.13** | **-0.11** | **-0.09** | **-0.19** | **0.05** | **0.17** |
|  | PIAT Read Comp | **0.09** | **-0.08** | **0.08** | **0.13** | **-0.11** | **-0.08** | **-0.06** | **-0.13** | **0.06** | **0.19** |
|  | PIAT Read Rec | **0.08** | **-0.07** | **0.07** | **0.12** | **-0.09** | **-0.07** | **-0.05** | **-0.10** | **0.07** | **0.19** |
| Relationships & Family | |  |  |  |  |  |  |  |  |  |  |
|  | Ever Married | **1.12** | 1.01 | **1.22** | **1.23** | **1.25** | **1.25** | 1.00 | 0.94 | **0.83** | **0.81** |
|  | Ever Divorced | 1.03 | 0.97 | 1.01 | **0.73** | 1.13 | 1.25 | **1.15** | 1.00 | 1.11 | 1.20 |
|  | Times Married | **1.08** | 1.01 | **1.13** | **1.15** | **1.18** | **1.17** | 1.01 | 0.96 | **0.89** | **0.88** |
|  | Rel. Satisfaction | **0.13** | 0.10 | **0.09** | 0.05 | **0.11** | -0.03 | **-0.19** | **-0.17** | -0.02 | -0.03 |
|  | Ever Children | 1.00 | 1.10 | 1.05 | 0.95 | **1.38** | **1.34** | **1.14** | **1.25** | **0.88** | **0.79** |
| Education, Career, & Financial | |  |  |  |  |  |  |  |  |  |  |
|  | Highest Degree | **0.17** | -0.03 | **0.12** | **0.31** | **0.08** | **0.13** | **-0.16** | **-0.19** | -0.04 | 0.07 |
|  | Employed | **1.15** | 1.03 | 1.01 | 1.12 | **1.09** | 1.11 | **0.89** | **0.83** | 1.04 | **1.19** |
|  | Ever Welfare | **0.77** | 0.90 | **1.21** | 0.94 | 0.98 | 0.88 | **1.34** | **1.46** | **0.83** | 0.85 |
|  | Annual Salary | **0.08** | **0.08** | **-0.03** | -0.02 | **0.07** | **0.06** | **-0.03** | **-0.05** | -0.01 | 0.03 |
| Civic Engagement | |  |  |  |  |  |  |  |  |  |  |
|  | Ever Volunteered | **1.30** | 1.13 | **1.13** | **1.28** | 1.03 | 1.12 | 0.95 | 0.89 | 1.06 | 1.11 |
|  | Religious | 1.10 | **1.54** | **1.23** | 1.20 | 1.11 | 1.19 | 1.08 | **1.75** | 0.97 | 1.20 |
| *Note*. W/O = without covariates. With = with covariates. Bolded values indicate that the credible intervals did not contain 1.00 (for *OR*s and *IRR*s) or 0.00 (for *b*s). See previous supplementary tables for the metric of all estimates per outcome (i.e., *OR*, *IRR*, or *b*). | | | | | | | | | | | |

**Figure S1**


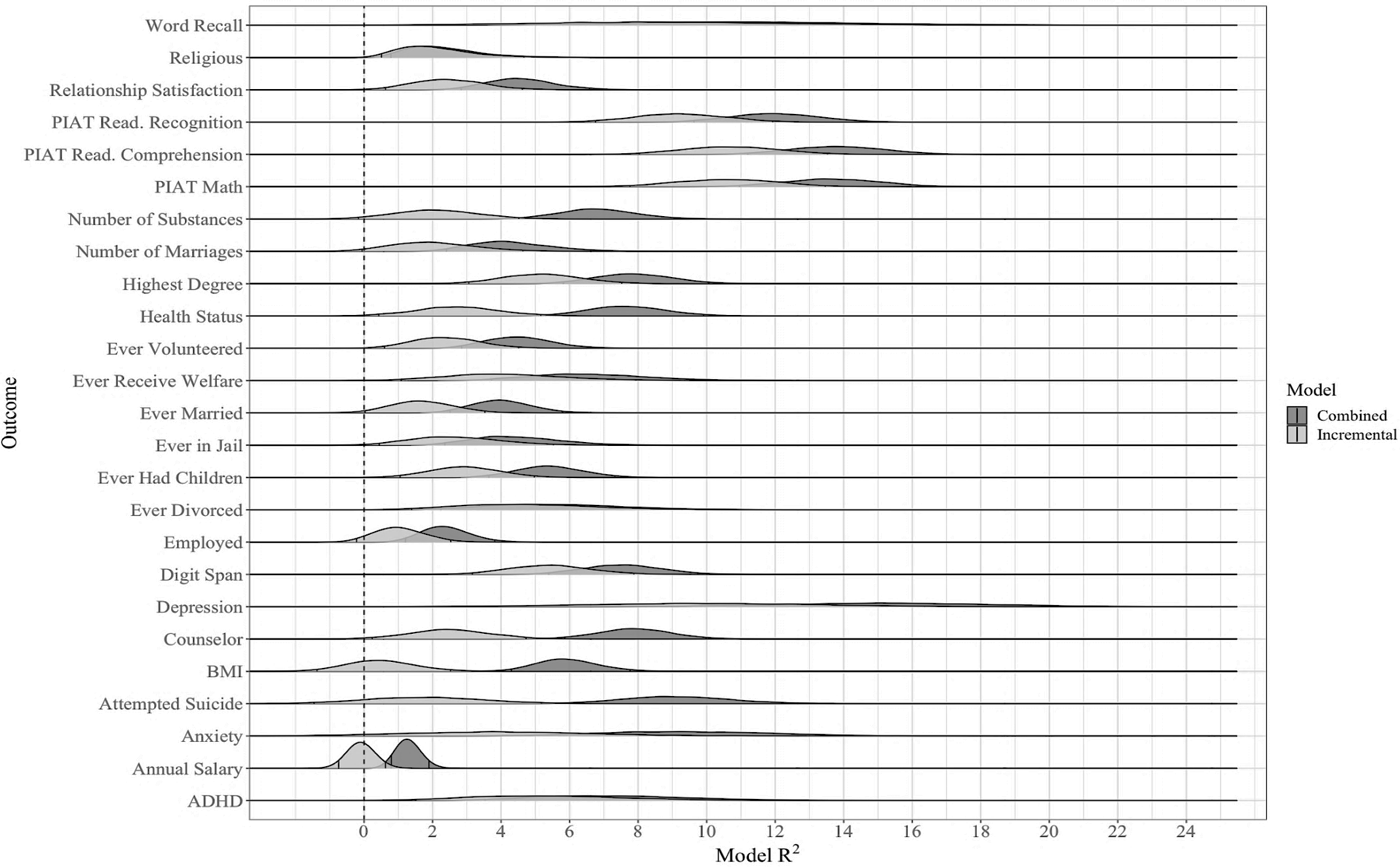
*R^2^ Distributions for the Combined Models and the Incremental Proportion of Variance Accounted for by Childhood Temperament for All Outcomes*

Figure S1. R^2^ values are presented as percentages. The R^2^ for the combined models (temperament & personality predictors) is plotted in dark gray. The portion of the combined R^2^ that has the R^2^ accounted for by the personality traits subtracted out is plotted in light gray (i.e., temperament’s incremental contribution). The 95% credible intervals, representing the R^2^ values that were present in 95% of the posterior distributions, are outlined in each distribution. To the extent that the light gray and dark gray overlap with each other, the temperament traits then account for most of the variance in predicting the respective outcome.
